# Response of geranium to soil applications of Azolla and Moringa extracts: effects on growth, essential oil and physiological parameters

**DOI:** 10.1186/s12870-026-09900-0

**Published:** 2026-09-21

**Authors:** Hala S. Abd-Rabbu, Fatma S. Aboud, Khalid A. Khalid

**Affiliations:** 1 Medicinal and Aromatic Plants Department, National Research Centre, Giza, Egypt; 2 Climate Change Information Center, Renewable Energy, Expert System, Agriculture Research Center, Giza, Egypt

**Keywords:** Mass production, Volatile constituents, Antioxidant, Chemical composition

## Abstract

**Background:**

Geranium (*Pelargonium graveolens* L.) is an important aromatic plant valued for its essential oil; however, its cultivation in sandy soils is often constrained by poor fertility and limited water-holding capacity. Therefore, this study was conducted to evaluate the effects of Azolla and Moringa extracts on growth, essential oil and physiological traits of geranium grown under sandy soil conditions.

**Methods:**

Azolla extract at concentrations of 50 and 75% and Moringa extract at 5 and 10% were supplied through irrigation water throughout the growing season, and the plants were harvested on two occasions. Data were statistically analyzed using 2 ways analysis of variance (ANOVA-2).

**Results:**

Obtained results demonstrated that both extracts enhanced morphological and physiological characteristics relative to the control treatment. Application of 10% Moringa extract produced the greatest improvement in all measured parameters compared with the untreated control: Plant height, branch number, and biomass (fresh and dry) increased by 2.5–2.6 fold, 2.1–2.3 fold and 2 -2.1 fold, respectively. Chlorophyll *a*, chlorophyll *b* and carotenoid increased by 1.7 fold, 2 fold and 1-1.3 fold, respectively. Essential oil content (%) and essential oil yield increased by 1.1–1.4 fold and 1.4–2.3 fold, respectively. Essential oil major components (citronellol, geraniol and α-gurjunene) and its major group (oxygenated monoterpene) increased by 2.4, 3.3, 2.7 and 2.3 fold, respectively. Phenols, flavonoids, inhibition of free radical, carbohydrate and crude protein increased by 1.5–1.6 fold, 1.6–1.9 fold, 1.7–1.8 fold, 2.3 fold and 1.5–1.8 fold, respectively. N, P and K content improved by 1.5–1.8 fold, 2.5 fold and 1.5–1.7 fold, respectively. N, P and K uptake increased by 3.1–3.6 fold, 5–25 folds and 2.9–3.3 fold, respectively.

**Conclusion:**

Overall, the application of Azolla or Moringa extracts proved to be an effective and environmentally friendly strategy for improving geranium performance and essential oil production in sandy soils. These natural biostimulants offer a sustainable alternative to synthetic inputs and may support the commercial cultivation of geranium in newly reclaimed and desert lands.

## Highlights

Azolla and Moringa soil applications enhance geranium productivity in sandy soil.

Azolla and Moringa act as eco-friendly biostimulants.

Both extracts reduce sandy soil stress by enhancing antioxidant activity.

Natural extracts provide a sustainable alternative to chemical fertilizers.

## Introduction

Aromatic plant cultivation has substantial economic potential owing to the increasing demand for their products in food, cosmetic, perfumery, pharmaceutical, and healthcare industries [1]. Aromatic plants are rich in essential oils with significant biological activities, and research suggests that some natural oils may be safer and less harmful than certain synthetic products under specific conditions [1]. Hence, promoting the cultivation of aromatic plant is important to enhance essential oil production for a wide range of applications [1]. Geranium (*Pelargonium graveolens* L.) is an evergreen perennial herb belonging to the Geraniaceae family and is native to the Mediterranean region [2]. It is widely cultivated for its essential oil, which is extensively used in the perfume, cosmetic, and aromatherapy industries due to its high content of geraniol, citronellol, and linalool [3, 4]. In addition to its commercial importance, geranium essential oil exhibits diverse biological properties, including antioxidant, antimicrobial, anti-inflammatory, anticancer, and antidiabetic activities, making it a valuable medicinal and aromatic crop [3, 4].

Adequate plant nutrition is essential for improving plant morphology and the accumulation of secondary metabolites in aromatic plants [5, 6]. Increasing restrictions on agrochemical use have encouraged the development of sustainable approaches to crop nutrition, with particular attention being given to naturally derived biostimulants [7]. Plant-based biostimulants contain a wide range of bioactive compounds, including phytohormones, vitamins, amino acids, and mineral nutrients, which enhance plant growth, productivity, and stress tolerance by regulating key physiological and metabolic processes [7, 8]. Consequently, their use has emerged as an environmentally friendly strategy for improving crop performance while reducing dependence on synthetic agrochemicals [9].

Sandy soils are highly susceptible to environmental stresses, particularly salinity and drought, because of their low water- and nutrient-holding capacity, which adversely affects the productivity and essential oil quality of aromatic plants [10, 11]. These conditions impair physiological and photosynthetic processes, increase oxidative stress, and ultimately reduce plant performance and essential oil yield [12–14]. Therefore, the application of biostimulants has emerged as a sustainable strategy to improve stress tolerance and enhance the productivity and quality of aromatic plants cultivated under sandy soil conditions [15, 16].

Azolla (*Azolla pinnata*) is a free-floating aquatic fern that forms a symbiotic association with the nitrogen-fixing cyanobacterium *Anabaena azollae*, enabling it to improve nitrogen availability and soil fertility [17]. Owing to its richness in nutrients, amino acids, vitamins, and natural growth-promoting compounds, Azolla has attracted considerable attention as a sustainable biostimulant for enhancing plant growth, nutrient uptake, and stress tolerance. Despite its promising potential, information regarding its application in medicinal and aromatic plants remains limited. To date, only one study has evaluated the effect of Azolla on marjoram, where its application improved growth, chlorophyll content, photosynthetic efficiency, essential oil yield, antioxidant activity, and stress tolerance under adverse conditions [18]. These findings highlight the need for further studies to evaluate the effectiveness of Azolla in other economically important aromatic species, such as geranium.

Moringa (*Moringa oleifera*) is widely recognized as a natural biostimulant because of its high content of essential nutrients, amino acids, vitamins, antioxidants, and phytohormones, particularly cytokinins and auxins, which promote plant growth, nutrient uptake, and stress tolerance [19]. Consequently, Moringa leaf extract has been successfully applied to improve the growth, productivity, physiological performance, and essential oil characteristics of several aromatic plants, including sage, damask rose, geranium, fennel, basil, cumin, and ajwain under both normal and stress conditions [20–26]. However, despite these promising findings, comparative information on the effectiveness of Moringa and Azolla extracts under sandy soil conditions, particularly across successive harvests of geranium, remains limited.

The ability of aromatic plants to be harvested several times during a single growing season represents an important agronomic advantage, increasing cumulative biomass and essential oil production from the same cultivation area. Harvest frequency is a key factor influencing plant productivity and quality because successive harvests alter physiological and metabolic processes, resulting in changes in biomass accumulation, essential oil yield and composition, photosynthetic pigments, primary metabolites, nutrient uptake, and secondary metabolites. These changes ultimately affect the phytochemical quality and antioxidant potential of aromatic plants. Therefore, optimizing harvest frequency is essential for maximizing yield and essential oil quality [27, 28].

Egypt’s cultivated land accounts for only about 4% of the country’s total area, making the reclamation of sandy soils a national priority. However, their low fertility and poor water-holding capacity limit the cultivation of aromatic plants, highlighting the need to enhance soil fertility and plant performance under these conditions [29, 30].

Although several studies have demonstrated the beneficial effects of Moringa extract on the productivity of aromatic plants, including geranium, limited information is available regarding the comparative performance of Azolla and Moringa extracts under sandy soil conditions. Moreover, little attention has been paid to their effects across successive harvests and their influence on growth, nutrient uptake, essential oil yield and composition and biochemical characteristics of geranium. Therefore, the present study was designed to compare the effectiveness of Azolla and Moringa extracts as natural biostimulants in improving productivity, physiological responses, nutrient uptake, and essential oil characteristics of geranium during two successive harvests under sandy soil conditions.

## Materials and methods

### Description of experimental procedures

Experiments were performed throughout two successive seasons in the years 2023 and 2024. Uniform geranium (*Pelargonium graveolens* L) seedlings were obtained from the experimental station at Al-Qanater Al-Khairia, affiliated with the Medicinal and Aromatic Plants Department, Horticulture Research Institute, Agricultural Research Center, Egypt. Seedlings were planted in plastic pots (30 cm diameter and 50 cm height) in the first week of March in both seasons. After 45 days, seedlings were thinned to three plants per pot and subsequently transferred to a greenhouse at the Central Laboratory for Agricultural Climate, Agricultural Research Center located at Giza Governorate, Egypt, where they were maintained throughout the experiment. Soil samples utilized in this study were characterized for their physical and chemical properties according to the procedures described by Jackson [31] and Cottenie [32], with the obtained data summarized in Table 1. On the other hand, the meteorological data of both seasons are recorded in Table 2.

**Table 1:** Physicochemical analysis of the experimental soil

Structural characteristics and components	Measures
Clay granules (%)	9.2
Sandy fraction (%)	83.5
Silt proportion (%)	7.3
Soil suspension pH (1: 2.5)	7.9
Electrolytic conductivity (dS m^− 1^)	1.4
Organic fraction (%)	0.1
Calcium carbonate (CaCO_3_) (%)	18.4
Nitrogen (N) level (%)	0.2
Phosphorus (P) value (mg 100 g^− 1^ Soil)	3.1
Cations (mg 100 g^− 1^)	
Potassium (K)	2.7
Iron (Fe)	0.8
Manganese (Mn)	1.2
Zinc (Zn)	2.2
Cupper (Cu)	2.4
Calcium (Ca)	22.7
Magnesium (Mg)	2.1
Sodium (Na)	39.3
Anionic components (mg 100 g^− 1^)	
Chloride (Cl)	22.4
Carbon trioxide (CO_2_)	22.3
Sulfate (SO_4_)	9.9
Nitrates (NO_3_)	2.9

**Table 2:** Meteorological data of growth seasons

Months	Temp. (°C)				RH (%)		WS (m/s)		SR (MJ/m²/d)	
	Seasons									
	2023		2024		2023	2024	2023	2024	2023	2024
	Min.	Max.	Min.	Max.						
March	12.1	12.8	26.6	26.8	53.0	51.5	2.5	2.4	19.6	19.9
April	14.7	15.6	30.1	31.2	48.3	45.1	2.7	2.6	23.3	23.8
May	18.2	19.1	34.6	35.3	43.1	42.8	2.8	2.8	26.4	27.1
June	21.6	22.8	37.1	38.2	43.7	44.3	3.0	2.9	28.3	28.6
July	24.1	24.5	39.8	39.5	45.2	48.7	2.7	2.6	28.5	27.9
August	24.4	24.8	39.1	39.7	49.8	51.2	2.5	2.4	26.1	25.8
September	21.9	21.5	36.5	36.1	53.4	55.6	2.6	2.5	21.9	21.2

*Temp. *Temperature, *Min. *Minimum, *Max. *Maximum, *RH *Relative Humidity, *WS *Wind Speed, *SR *Solar Radiation

In this study, five different treatments were applied to the plants. The control treatment (T1) was irrigated only with the complete nutrient solution according to Hoagland [33]. The nutrient solution contained the following essential elements: N (210 mg L^-1^), K (235 mg L^-1^), Ca (200 mg L^-1^), P (31 mg L^-1^), S (64 mg L^-1^), Mg (48 mg L^-1^), B (0.5 mg L^-1^), Fe (1–5 mg L^-1^), Mn (0.5 mg L^-1^), Zn (0.05 mg L^-1^), Cu (0.02 mg L^-1^), and Mo (0.01 mg L^-1^). In contrast, other treatments were irrigated with aqueous extract of Azolla or Moringa extract. The experimental treatments were designated as follows: T1 served as the control and received the complete nutrient solution only. T2 and T3 received Azolla extract at concentrations of 50 and 75%, respectively, applied through the irrigation water. T4 and T5 received Moringa extract at concentrations of 5 and 10%, respectively, applied through the irrigation water. Azolla and Moringa extracts were applied weekly through irrigation water after thinning throughout the growing season at the designated concentrations to ensure uniform distribution in the root zone and efficient plant uptake.

### Preparation of Azolla extract

Following the procedure described by Yoshida [34], One kg of washed fresh Azolla biomass was boiled in 1 L of distilled water for 30 min. After cooling to room temperature, the extract was filtered through a double layer of muslin cloth. The resulting filtrate was considered the stock extract (100% concentration). Subsequently, diluted extracts of 75% and 50% were prepared from the stock solution using distilled water. All extracts were stored under refrigeration (0–4 °C) until use. A fresh extract was prepared before each application to ensure consistency and minimize changes in extract composition. Composition of Azolla extract is presented in Table 3.

**Table 3:** Characterization of Moringa leaves

Attributes	Index
Phenols (mg g^− 1^)	19.3
Flavonoids (mg g^− 1^)	21.7
Tannin (mg 100 g^− 1^)	0.42
Phytic acid (mg 100 g^− 1^)	0.1
Carotene (mg g^− 1^)	0.9
Lycopene (mg g^− 1^)	0.5
Carbohydrates (g 100 g^− 1^)	31.2
Lipid (g 100 g^− 1^)	6.9
Protein (g 100 g^− 1^)	25.3
Amino acids (mg 100 g^− 1^)	82.3
Ascorbic acid (mg 100 g^− 1^)	598
Antioxidant activity (%)	82.6
Dietary fiber (g 100 g^− 1^)	26.2
Ash (g 100 g^− 1^)	10.2
Ca (%)	2.3
K (%)	1.3
Mg (%)	0.7
Na (%)	0.1
P (%)	0.3
Cu (ppm)	7.5
Fe (ppm)	118.4
Zn (ppm)	31.7
Mn (ppm)	77.4

### Preparation of Moringa extract

Moringa leaf extract was prepared at the Central Laboratory for Agricultural Climate (CLAC), Agricultural Research Center, according to the method described by Foidl [35] and Yasmeen [19]. Fresh Moringa leaves were blended with distilled water at a ratio of 1 kg of fresh leaves per liter of water using a locally fabricated extraction machine. The crude extract was filtered through a double layer of muslin cloth and then centrifuged at 8,000 × g for 15 min to remove solid residues. The resulting supernatant was collected and used as the stock solution. Subsequently, the stock extract was diluted with distilled water to obtain final concentrations of 5% and 10% (v/v). A fresh extract was prepared before each application. The chemical composition of the prepared extract is shown in Table 4.

**Table 4:** Characterization of Azolla

Attributes	Index
Phenols (mg g^− 1^)	24.6
Flavonoids (mg g^− 1^)	11.8
Photosynthetic pigments (mg g^− 1^)	2.95
Antioxidant (%)	72.5
Amino acids (g 100 g^− 1^)	41.6
Moisture (%)	91.2
Dry matter (%)	7.8
Crude protein (%)	27.9
Crude fiber (%)	13.5
Crude lipids (%)	4.6
Carbohydrates (%)	38.6
Ash (%)	14.4
N (%)	4.5
P (%)	0.6
K (%)	2.5
Ca (%)	1.3
Mg (%)	0.4
Fe (mg kg⁻¹)	1846.4
Zn (mg kg⁻¹)	47.4
Cu (mg kg⁻¹)	14.3
Mn (mg kg⁻¹)	126.4

### Shoot biomass evaluation

Biomass collection was performed twice during the growing season at 90 and 180 days after planting. The aerial parts of the plants were harvested by cutting the shoots approximately 5 cm above the soil surface to allow subsequent regrowth. Fresh weight of harvested herb was recorded immediately after cutting, while dry weight (g plant^− 1^) was determined after drying the samples to a constant weight. The obtained fresh and dry weight values represented the average performance of plants in each growing season.

### Spectrophotometric analysis of photosynthetic pigments

The AOAC [36] standard procedure was followed to determine the concentrations of photosynthetic pigments, including chlorophyll *a*, chlorophyll *b* and total carotenoids, in fresh geranium leaves. Fresh leaf tissues were homogenized in 80% acetone using a mortar and pestle until a uniform extract was obtained. The extract was then filtered, and its absorbance was measured spectrophotometrically using a Shimadzu UV-1700 instrument (Tokyo, Japan) at 662 nm and 645 nm for chlorophyll a and b, respectively, and at 470 nm for total carotenoids. Pigment contents were calculated and expressed as milligrams per gram of fresh weight (mg g^− 1^ FW).

### Hydro-distillation of essential oil

In each season, fresh aerial plant material was collected. From each replicate (three replicates), 250 g of finely chopped fresh material was mixed with 1000 mL of distilled water in a 2 L round-bottom flask and subjected to hydro-distillation using a Clevenger-type apparatus for 3 h at 100 °C, or until no further essential oil was recovered [37]. The extracted essential oil was collected, separated from the aqueous phase, and stored for subsequent GC–MS analysis. Essential oil content was expressed as a percentage on a weight-to-weight basis (w/w) and essential oil yield was calculated according to dry weight (g plant^− 1^).

### Analysis of essential oil

Gas chromatographic (GC) analyses were conducted using a Shimadzu GC-9 gas chromatograph fitted with a DB-5 fused silica capillary column (5% phenyl dimethylsiloxane; J & W Scientific Corporation) measuring 60 m × 0.25 mm i.d. with a film thickness of 0.25 μm. The oven temperature was initially maintained at 50 °C for 5 min, and then increased to 240 °C at a rate of 3 °C min^− 1^. The injector and flame ionization detector (FID) temperatures were set at 250 °C and 265 °C, respectively. Helium served as the carrier gas at a linear flow velocity of 32 cm s^− 1^. Compound percentages were determined using the area normalization method without applying correction factors. GC/MS analyses were performed using a Varian 3400 GC–MS system equipped with a DB-5 fused silica capillary column (60 m × 0.25 mm i.d., film thickness 0.25 μm). The oven temperature program ranged from 50 to 240 °C at a heating rate of 3 °C min^− 1^, while the transfer line temperature was maintained at 260 °C. Helium was employed as the carrier gas at a linear velocity of 31.5 cm s⁻¹, with a split ratio of 1:60. Mass spectra were recorded under electron ionization (EI) conditions at 70 eV, using a scan time of 1 s and a mass range of 40–300 amu.

### Identification of volatile constituents

Essential oil constituents were identified by comparing their mass spectra with those of authentic reference compounds and the NIST/NBS, Wiley 275.L, and Mass Finder 3 mass spectral libraries. Identification was further confirmed by comparing the calculated retention indices (RI), determined using a homologous series of n-alkanes (C8–C22) under identical chromatographic conditions, with published literature data and authentic standards [38]. Quantitative analysis was based on the relative percentage peak areas obtained from the flame ionization detector (FID).

### Measurement of phenols

Total phenolic content in leaf samples was estimated using the Folin–Ciocalteu colorimetric assay following the method of Singleton [39]. Briefly, 200 µL of crude extract (1 mg/mL) was diluted with distilled water to obtain a final volume of 3 mL. The diluted extract was then reacted with 0.5 mL of Folin–Ciocalteu reagent and allowed to stand for 3 min to ensure initial reaction. Afterwards, 2 mL of sodium carbonate solution (20% w/v) were added to the mixture. The reaction was kept in complete darkness for 60 min at room conditions to allow full color development. Finally, the absorbance of the resulting solution was measured at 650 nm using a spectrophotometer. Total phenolics were quantified from a gallic acid standard calibration curve and expressed as mg gallic acid equivalents per gram of dry weight (mg GAE/g DW).

### Estimation of flavonoids

Total flavonoid content in leaf samples was determined using the aluminum chloride colorimetric method as described by Pourmorad [40]. Briefly, 50 µL of crude extract (1 mg/mL in ethanol) was mixed with 1 mL of methanol and diluted with 4 mL of distilled water. Then, 0.3 mL of 5% sodium nitrite solution was added, and the mixture was allowed to stand for 5 min. Subsequently, 0.3 mL of 10% aluminum chloride solution was introduced, followed by incubation for 6 min. After that, 2 mL of 1 mol/L sodium hydroxide was added, and the final volume was adjusted to 10 mL with double-distilled water. The solution was left to stand for 15 min, and the absorbance was measured at 510 nm using a spectrophotometer. Total flavonoid content was calculated from a rutin standard calibration curve and expressed as milligrams of rutin equivalents per gram of dry weight (mg RE/g DW).

### Analysis of antioxidant capacity using free radical scavenging assay

The antioxidant potential of the leaf extracts was determined using the 1, 1-diphenyl-2-picrylhydrazyl (DPPH) free radical scavenging assay as described by Middha [41]. In this method, 200 µL of each extract at graded concentrations (100–500 µg/mL) was added to 3.8 mL of freshly prepared DPPH solution. The reaction mixtures were thoroughly mixed and incubated in the dark at room temperature for 60 min to allow completion of the reaction. The absorbance was subsequently measured at 517 nm using a spectrophotometer. Ascorbic acid was employed as a standard antioxidant for comparison. The scavenging ability of the extracts was estimated based on the reduction in DPPH absorbance, indicating their capacity to donate hydrogen atoms or electrons and neutralize free radicals.

### Assessment of carbohydrates

Total carbohydrates content in dry leaf samples was determined using the phenol–sulfuric acid colorimetric method of Dubois [42]. In brief, carbohydrates were extracted by homogenizing the dried leaf material in 1 N H2SO_4_ to obtain a clear extract. The reaction was developed by treating the extract under the standard colorimetric conditions, and the resulting color intensity was measured spectrophotometrically at 490 nm. D-glucose was used as a reference standard for constructing the calibration curve. Total carbohydrate levels were calculated from the standard curve and expressed on a dry weight basis.

### Estimation of crude protein

Crude protein content in leaf samples was determined using the micro-Kjeldahl method according to AOAC [36]. Briefly, 1 g of oven-dried plant material was digested with 15 mL of concentrated sulfuric acid in the presence of two copper catalyst tablets using a Kjeltec System 2020 digestor (Tecator Inc., Herndon, VA, USA) at 420 °C for 2 h. After digestion and cooling, the mixture was diluted with distilled water, then subjected to neutralization and distillation, followed by titration to estimate total nitrogen content. Crude protein content was subsequently calculated by multiplying the total nitrogen percentage by the standard conversion factor of 6.25.

### Assessment of elemental constituents

Elemental composition of leaf samples was determined following the method described by Jackson [31]. Briefly, 0.5 g of finely powdered leaf material was transferred into Teflon PFA digestion vessels and treated with a concentrated acid mixture of HNO3: HCl (3:1). The samples were digested at 85 °C for 3 h, and 1.0 mL of concentrated HClO was added during the digestion process to enhance oxidation. After complete digestion, the solutions were filtered and diluted to a final volume of 50 mL with distilled water. Blank samples were prepared simultaneously using the same reagents without plant material. Nitrogen (N) content was determined using the micro-Kjeldahl method according to AOAC [36], phosphorus (P) was measured spectrophotometrically following the method of Snell [43], and potassium (K) concentration was estimated using a flame photometer.

### Experimental design and statistical procedures

The experiment was arranged in a completely randomized design (CRD) with four replicates per treatment. Each replicate consisted of eight pots, with three plants grown in each pot. The study was conducted as a two-factor factorial experiment involving five application treatments evaluated at two harvests. The combined mean data of the two growing seasons were subjected to two-way analysis of variance (ANOVA-2) following the procedure described by Snedecor [44]. The effects of treatments, harvests, and their interactions were assessed based on the calculated mean values, and significance among treatments was determined according to *P*-values. Statistical analyses were carried out using the STAT-ITCF software package as described by Foucart [45]. All values were given as mean ± standard divination (SD).

## Results

### Different applications, harvests, and their combinations’ effects on growth characters

Table 5 displays the effects of different applications, harvests, and their interactions on the plant height, branch number, fresh and dry mass for both seasons. Plants collected at the second harvest resulted in higher growth characters than those collected at first harvest of both seasons. Changes in various applications during both harvests have a positive impact on different growth characters. Consequently, Azolla and Moringa extracts significantly enhanced the vegetative growth of geranium plants, with the greatest response obtained from the 10% Moringa extract. This treatment produced the highest values of plant height (114.5 and 119.6 cm), number of branches (8.0 and 10.3 plant⁻¹), fresh mass (685.3 and 677.7 g plant⁻¹), and dry mass (119.9 and 126 g plant⁻¹) at the second harvest during the first and second seasons, respectively. Relative to the control, the 10% Moringa extract increased plant height by 2.5- and 2.6-fold during the first and second seasons, respectively. Similarly, the number of branches increased by 2.1–2.3 fold, while fresh and dry weights increased by 2- 2.1-fold, respectively. The variations observed in different growth characters were highly significant (*P ≤ 0.001*) in response to application treatments, harvests, and their interactions. However, the interaction effect on branch number in the second season was significant (*P ≤ 0.05*), while no significant interaction effects were detected for branch number during the first season or for fresh and dry mass during the second season.

**Table 5:** Effect of Azolla and Moringa extracts on growth parameters during both harvests

Collections	Applications(%)		Plant height (cm)		Branch number (Plant^-1^)		Mass production (g plant^-1^)			
							Fresh		Dry	
			Seasons							
			1^st^	2^nd^	1^st^	2^nd^	1^st^	2^nd^	1^st^	2^nd^
First	Control		44.5 ± 4.4	48.8 ± 3.2	3.3 ± 0.5	4.7 ± 0.4	323.4 ± 7.6	343.4 ± 7.6	56.3 ± 3.6	62.5 ± 3.6
	Azolla	50	47.8 ± 3.8	45.2 ± 2.9	4.3 ± 0.6	5.0 ± 0.5	359.4 ± 6.9	374.8 ± 4.7	65.4 ± 4.6	70.1 ± 7.4
		75	50.0 ± 3.7	52.0 ± 3.7	4.7 ± 0.4	6.0 ± 0.7	379.9 ± 8.4	418.1 ± 8.4	71.4 ± 5.3	80.3 ± 4.3
	Moringa	5	50.6 ± 4.6	60.4 ± 3.7	5.3 ± 0.6	6.3 ± 0.3	433.9 ± 5.9	457.1 ± 10.5	82.4 ± 4.7	89.6 ± 6.3
		10	58.0 ± 4.8	63.0 ± 3.7	6.3 ± 0.7	6.7 ± 0.5	475.9 ± 8.9	506.1 ± 9.2	92.8 ± 3.7	100.2 ± 7.3
Overall first			50.2 ± 6.3	53.9 ± 3.8	4.8 ± 0.5	5.7 ± 0.8	394.5 ± 7.8	419.9 ± 5.7	73.72.7	80.5 ± 4.3
Second	Control		87.5 ± 6.7	90.3 ± 4.2	6.3 ± 0.4	5.7 ± 0.9	506.3 ± 9.6	530.3 ± 9.3	79.0 ± 4.2	86.4 ± 6.4
	Azolla	50	94.3 ± 6.8	101.3 ± 4.6	6.7 ± 0.4	7.3 ± 0.5	538.3 ± 8.5	553.4 ± 7.5	86.1 ± 6.4	96.3 ± 4.8
		75	100.9 ± 7.3	109.0 ± 4.8	6.7 ± 0.5	8.7 ± 0.8	574.0 ± 6.5	593.5 ± 8.1	94.1 ± 3.2	106.2 ± 7.3
	Moringa	5	112.5 ± 6.4	113.4 ± 3.6	7.3 ± 0.5	9.3 ± 0.5	622.0 ± 6.9	660.4 ± 5.7	105.1 ± 9.3	118.9 ± 6.5
		10	114.5 ± 7.3	119.6 ± 4.3	8.0 ± 0.7	10.3 ± 1.1	685.3 ± 7.5	677.7 ± 4.8	119.9 ± 7.9	126.0 ± 4.7
Overall second			101.9 ± 5.9	106.7 ± 5.7	7.0 ± 0.5	8.3 ± 0.9	585.2 ± 6.9	603.1 ± 6.7	96.9 ± 6.9	106.8 ± 8.7
Overall Applications	Control		66.0 ± 4.1	69.6 ± 3.7	4.8 ± 0.6	5.2 ± 0.4	414.9 ± 8.5	436.9 ± 4.9	67.6 ± 7.9	74.5 ± 3.2
	Azolla	50	71.1 ± 3.9	73.3 ± 3.8	5.5 ± 0.4	6.2 ± 0.5	448.9 ± 8.3	464.1 ± 8.4	75.8 ± 7.2	83.2 ± 5.8
		75	75.5 ± 4.7	80.5 ± 5.7	5.7 ± 0.8	7.3 ± 0.6	476.9 ± 9.5	505.8 ± 8.5	82.8 ± 4.8	93.3 ± 5.2
	Moringa	5	81.6 ± 4.9	86.9 ± 5.7	6.3 ± 0.6	7.8 ± 0.4	527.9 ± 6.5	558.8 ± 9.3	93.8 ± 6.2	104.2 ± 7.3
		10	86.3 ± 5.1	91.3 ± 5.1	7.2 ± 0.8	8.5 ± 0.7	580.6 ± 5.8	591.9 ± 8.9	106.4 ± 8.6	113.1 ± 8.4
F values										
Collections			5102.4^***^	18435.8^***^	90.8^***^	103.1^***^	32297.3^***^	2138.8^***^	4489.3^***^	1257.3^***^
Applications			99.3^***^	436.0^***^	11.8^***^	22.9^***^	390.6^***^	210.9^***^	1543.7^***^	353.6^***^
Collections x Applications			21.9^***^	56.1^***^	1.0 ^ns^	3.1^*^	183.3^***^	2.0 ^ns^	9.3^***^	1.3 ^ns^

*ns *non-significant

***, *P* ≤ 0.001; values are given as mean ± SD

### Reaction of photosynthetic pigments to different applications at various harvests

Photosynthetic pigments including Chlorophyll *a*, Chlorophyll *b*, and carotenoids increased in plants exposed to Azolla and Moringa extracts at both harvests in both seasons (Table 6). Higher values were found in photosynthetic pigments at first harvest compared with second harvest. Plants subjected to 10% Moringa extract at second harvest resulted in the greatest amounts of Chlorophyll *a*, Chlorophyll *b*, and carotenoids with the values of 0.8 and 0.9 mg g^− 1^; 0.4 and 0.5 mg g^− 1^; 0.6 and 0.6 mg g^− 1^ at the first and second seasons respectively. Compared with the control, treatment with 10% Moringa extract resulted in a pronounced enhancement of photosynthetic pigments. Chlorophyll *a* increased by 1.7 fold in both seasons, whereas chlorophyll *b* and carotenoid contents increased by 2 fold and 1 to 1.3 fold, respectively. Chlorophyll *a* content exhibited highly significant variations (*P ≤ 0.001*) due to the effects of application treatments and harvests, whereas their interaction had no significant influence. Chlorophyll *b* content showed significant differences (*P ≤ 0.05*) as affected by application treatments and harvests, while the interaction between both factors exerted no significant effect. Carotenoid content was moderately significantly affected (*P ≤ 0.01*) by the application treatments, while neither harvests nor the interaction between treatments and harvests showed significant effects.

**Table 6:** Effect of Azolla and Moringa extracts on photosynthetic pigments and essential oil output at both harvests

Collections	Applications(%)		Photosynthetic pigments (mg g^-1^)						Essential oil output			
			Ch *a*		Ch *b*		Carotenoids		(%)		(g plant^-1^)	
			Seasons									
			1^st^	2^nd^	1^st^	2^nd^	1^st^	2^nd^	1^st^	2^nd^	1^st^	2nd
First	Control		0.6 ± 0.2	0.7 ± 0.1	0.3 ± 0.1	0.3 ± 0.1	0.4 ± 0.1	0.4 ± 0.1	1.1 ± 0.3	1.2 ± 0.1	0.6 ± 0.1	0.8 ± 0.1
	Azolla	50	0.7 ± 0.2	0.7 ± 0.2	0.3 ± 0.1	0.3 ± 0.1	0.5 ± 0.2	0.4 ± 0.2	1.2 ± 0.2	1.3 ± 0.4	0.8 ± 0.3	0.9 ± 0.2
		75	0.7 ± 0.1	0.8 ± 0.3	0.4 ± 0.2	0.4 ± 0.1	0.5 ± 0.1	0.5 ± 0.2	1.3 ± 0.2	1.4 ± 0.3	0.9 ± 0.2	1.1 ± 0.3
	Moringa	5	0.8 ± 0.3	0.9 ± 0.2	0.4 ± 0.2	0.4 ± 0.2	0.5 ± 0.1	0.6 ± 0.3	1.4 ± 0.4	1.3 ± 0.4	1.1 ± 0.1	1.2 ± 0.4
		10	0.8 ± 0.2	0.9 ± 0.1	0.4 ± 0.2	0.4 ± 0.1	0.6 ± 0.2	0.6 ± 0.1	1.4 ± 0.3	1.4 ± 0.3	1.2 ± 0.4	1.4 ± 0.3
Overall first			0.7 ± 0.1	0.8 ± 0.2	0.3 ± 0.1	0.4 ± 0.2	0.5 ± 0.2	0.5 ± 0.2	1.3 ± 0.13	1.3 ± 0.4	0.9 ± 0.2	1.1 ± 0.2
Second	Control		0.8 ± 0.2	0.8 ± 0.2	0.3 ± 0.1	0.4 ± 0.1	0.5 ± 0.1	0.5 ± 0.2	1.1 ± 0.1	1.1 ± 0.2	0.9 ± 0.1	0.9 ± 0.3
	Azolla	50	0.8 ± 0.2	0.8 ± 0.1	0.4 ± 0.2	0.4 ± 0.1	0.5 ± 0.1	0.5 ± 0.2	1.1 ± 0.3	1.1 ± 0.3	0.9 ± 0.3	1.1 ± 0.4
		75	0.8 ± 0.2	0.9 ± 0.1	0.4 ± 0.2	0.5 ± 0.2	0.6 ± 0.3	0.6 ± 0.2	1.1 ± 0.2	1.2 ± 0.3	1.0 ± 0.3	1.3 ± 0.3
	Moringa	5	0.9 ± 0.3	0.9 ± 0.3	0.5 ± 0.1	0.5 ± 0.1	0.6 ± 0.3	0.6 ± 0.2	1.2 ± 0.4	1.2 ± 0.5	1.2 ± 0.3	1.5 ± 0.3
		10	1.0 ± 0.3	1.0 ± 0.3	0.6 ± 0.3	0.6 ± 0.3	0.7 ± 0.2	0.7 ± 0.1	1.5 ± 0.4	1.3 ± 0.3	1.4 ± 0.3	1.7 ± 0.5
Overall second			0.8 ± 0.1	0.9 ± 0.2	0.4 ± 0.1	0.5 ± 0.2	0.6 ± 0.2	0.6 ± 0.1	1.2 ± 0.3	1.2 ± 0.3	1.1 ± 0.4	1.3 ± 0.3
Overall Applications	Control		0.7 ± 0.2	0.7 ± 0.3	0.3 ± 0.1	0.3 ± 0.1	0.4 ± 0.1	0.4 ± 0.1	1.1 ± 0.3	1.2 ± 0.2	0.8 ± 0.2	0.9 ± 0.1
	Azolla	50	0.7 ± 0.2	0.8 ± 0.1	0.3 ± 0.1	0.4 ± 0.1	0.5 ± 0.2	0.5 ± 0.2	1.2 ± 0.4	1.2 ± 0.1	0.9 ± 0.3	1.0 ± 0.2
		75	0.8 ± 0.3	0.9 ± 0.4	0.4 ± 0.1	0.4 ± 0.1	0.6 ± 0.2	0.5 ± 0.1	1.2 ± 0.3	1.3 ± 0.1	1.0 ± 0.2	1.2 ± 0.3
	Moringa	5	0.8 ± 0.1	0.9 ± 0.2	0.4 ± 0.2	0.5 ± 0.2	0.7 ± 0.2	0.6 ± 0.1	1.3 ± 0.3	1.3 ± 0.3	1.2 ± 0.3	1.4 ± 0.4
		10	0.9 ± 0.2	1.0 ± 0.3	0.6 ± 0.3	0.5 ± 0.2	0.6 ± 0.2	0.7 ± 0.1	1.4 ± 0.4	1.4 ± 0.4	1.3 ± 0.4	1.6 ± 0.4
F values												
Collections			271.7^***^	371.6^***^	3.1^*^	16.2^*^	1.0 ^ns^	2.7 ^ns^	158.7^***^	93.1^***^	9.8^***^	11.8^***^
Applications			123.8^***^	234.6^***^	3.2^*^	5.3^*^	83.7^**^	7.0^**^	34.8^***^	29.1^***^	16.4^***^	12.3^***^
Collections x Applications			1.3 ^ns^	1.2 ^ns^	0.3 ^ns^	0.6 ^ns^	3.5 ^ns^	0.3 ^ns^	1.2 ^ns^	1.6 ^ns^	0.2 ^ns^	0.2 ^ns^

*ns *non-significant

***, *P* ≤ 0.001; **, *P* ≤ 0.01; *, *P* ≤ 0.05; values are given as mean ± SD

### Effects of various applications on essential oil production during different harvests

Essential oil content (%) and yield increased in plants treated with Azolla and Moringa extracts at both harvests (Table 6). Essential oil content was higher during the first harvest compared with the second harvest, whereas essential oil yield exhibited the opposite trend, recording greater values in the second harvest. Plants treated with 10% Moringa extract at second harvest had the highest level of essential oil content (1.5 and 1.3%) and essential oil yield (1.4 and 1.7 g plant^− 1^). Essential oil content (%) increased by 1.1–1.4 fold in both seasons, whereas essential oil yield increased by 1.4–2.3 fold at both seasons by this treatment. Essential oil production exhibited highly significant variation (*P ≤ 0.001*) under the influence of application treatments and harvests, while no significant effect was detected for their interaction.

### Influence of applications and harvests and their interactions on essential oil constituents

**Fig. 1:**
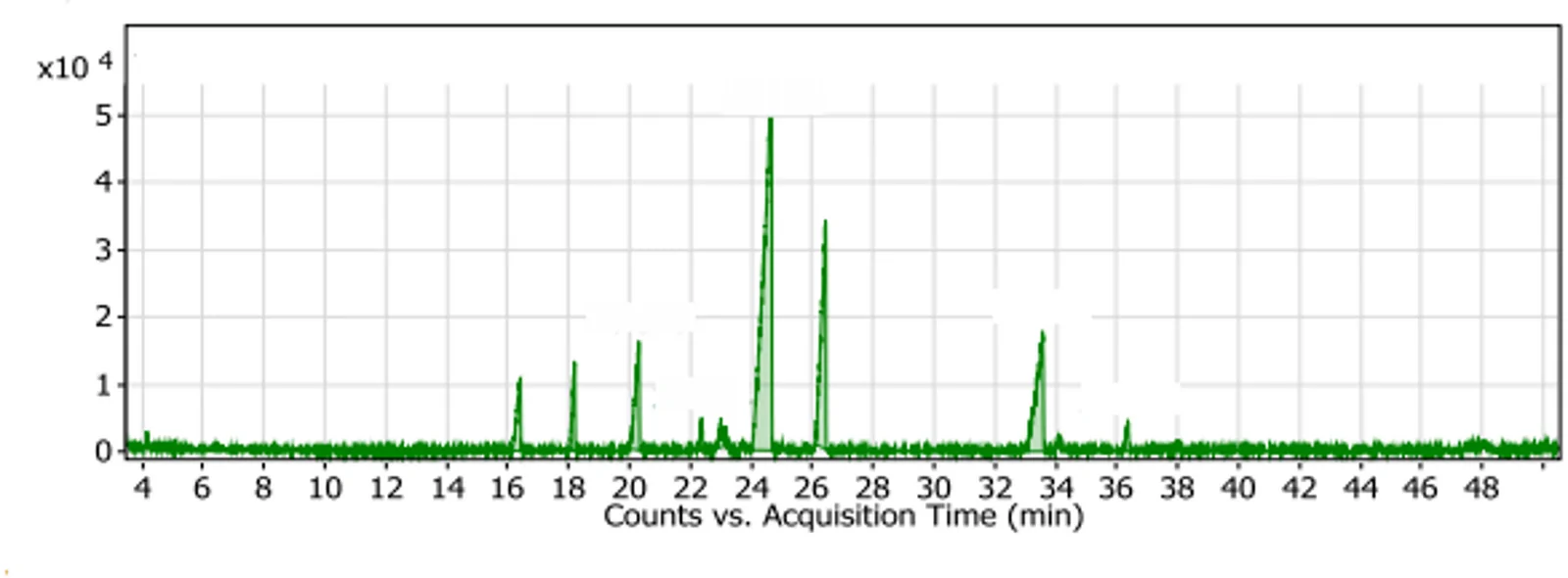
Essential oil constitutes from control treatments at first harvest

**Fig. 2:**
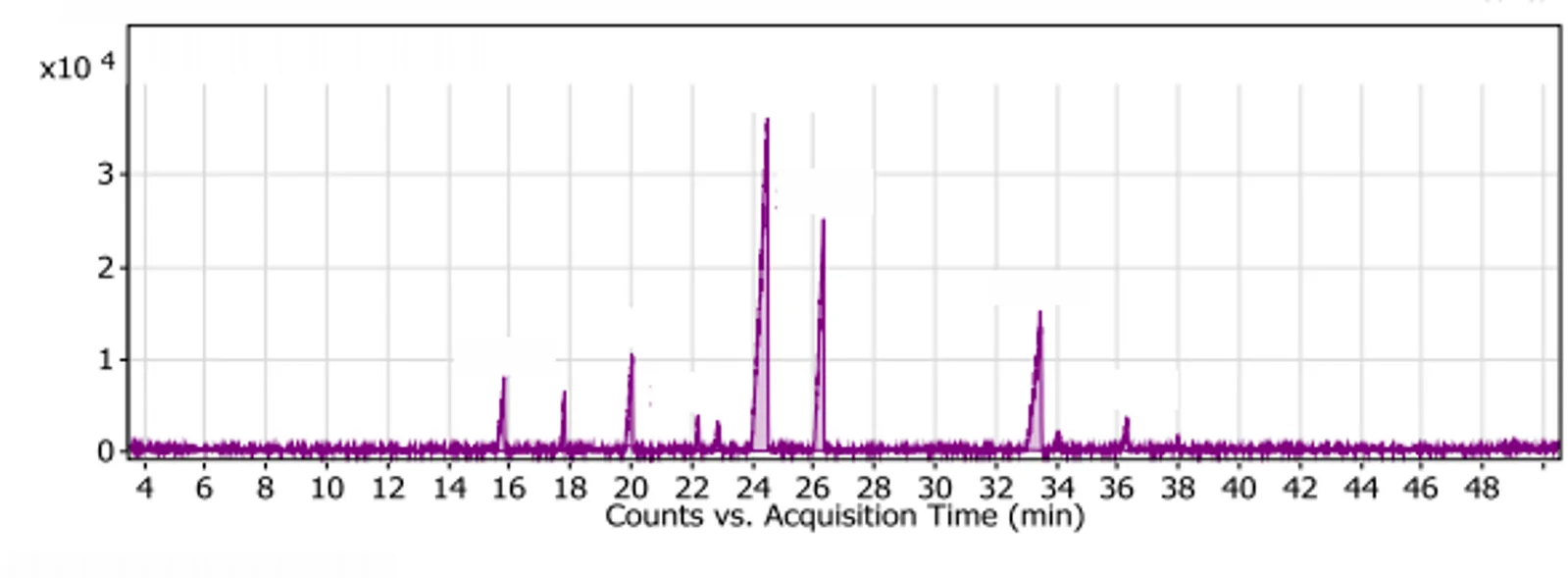
Essential oil constitutes from Azolla extract treatment (50%) at first harvest

**Fig. 3:**
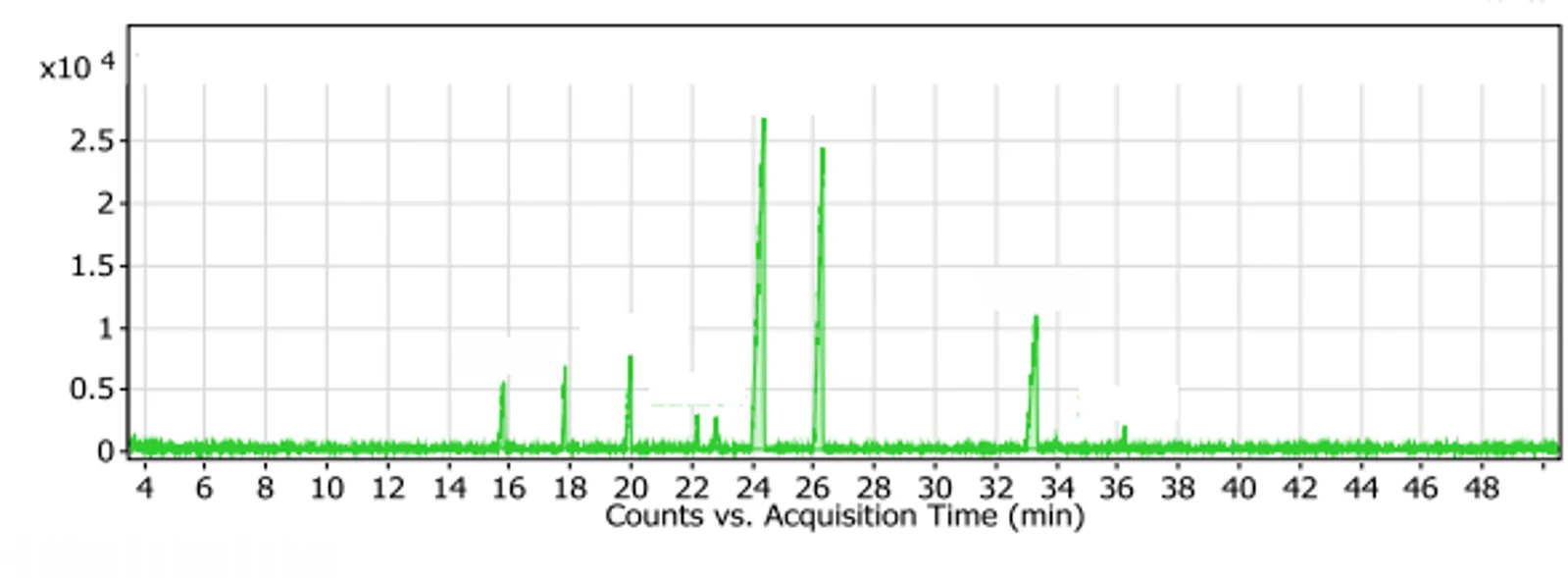
Essential oil constitutes from Azolla extract treatment (75%) at first harvest

**Fig. 4:**
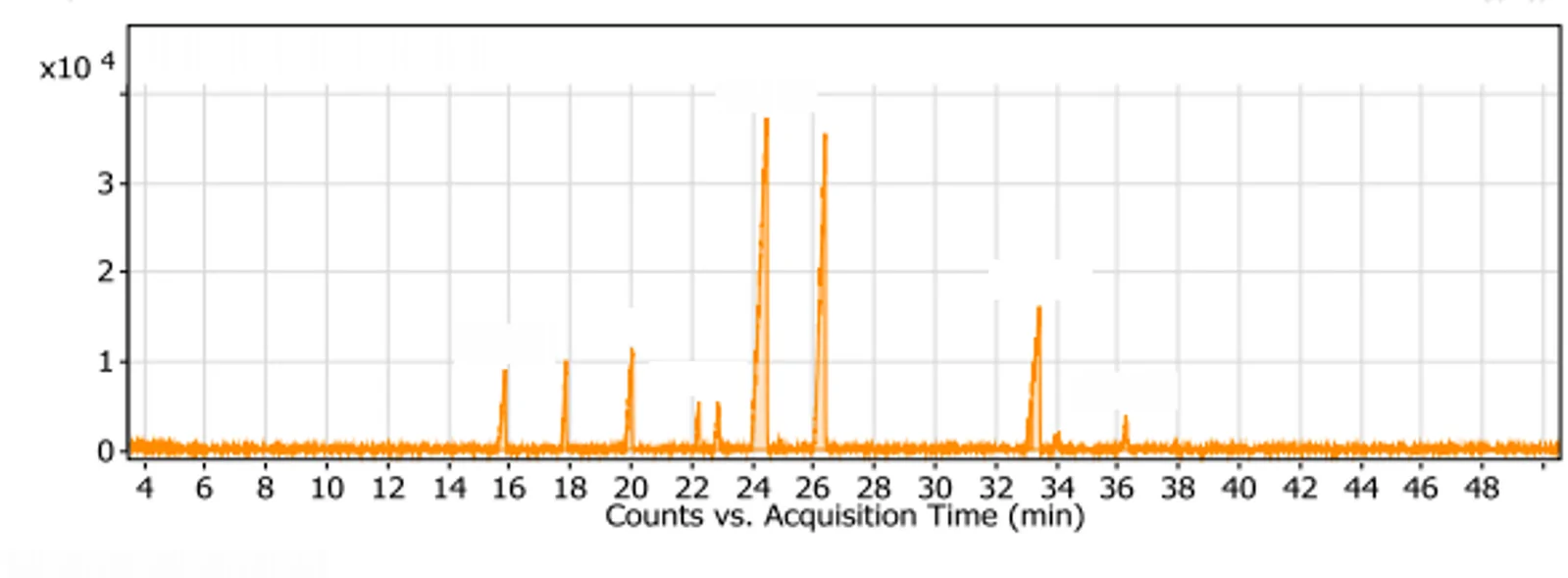
Essential oil constitutes from Moringa extract treatment (5%) at first harvest

**Fig. 5:**
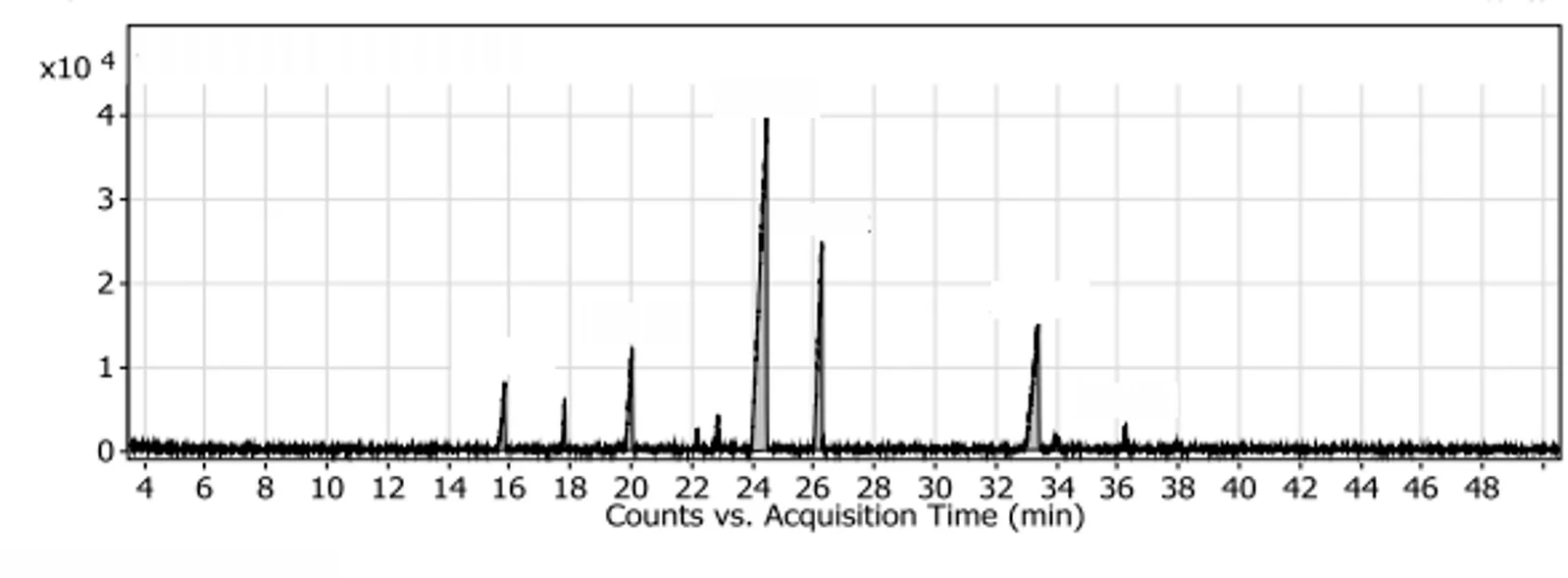
Essential oil constitutes from Moringa extract treatment (10%) at first harvest

**Fig. 6:**
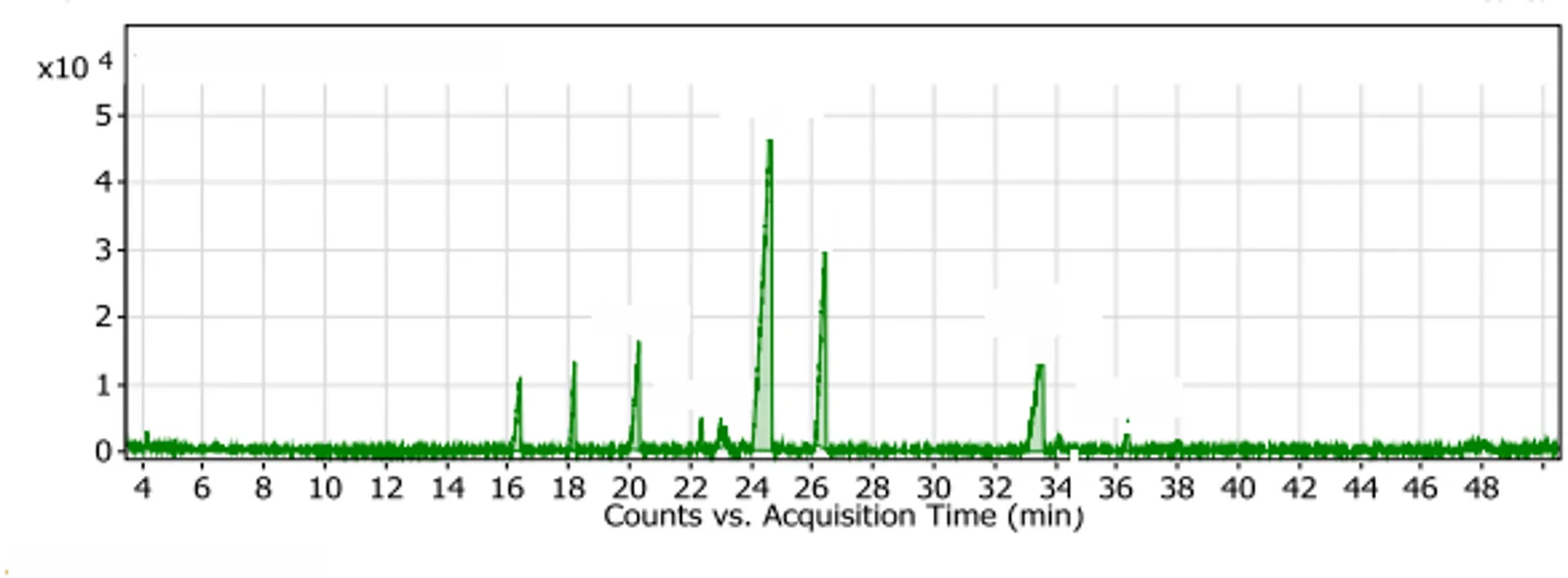
Essential oil constitutes from control treatments at second harvest

**Fig. 7:**
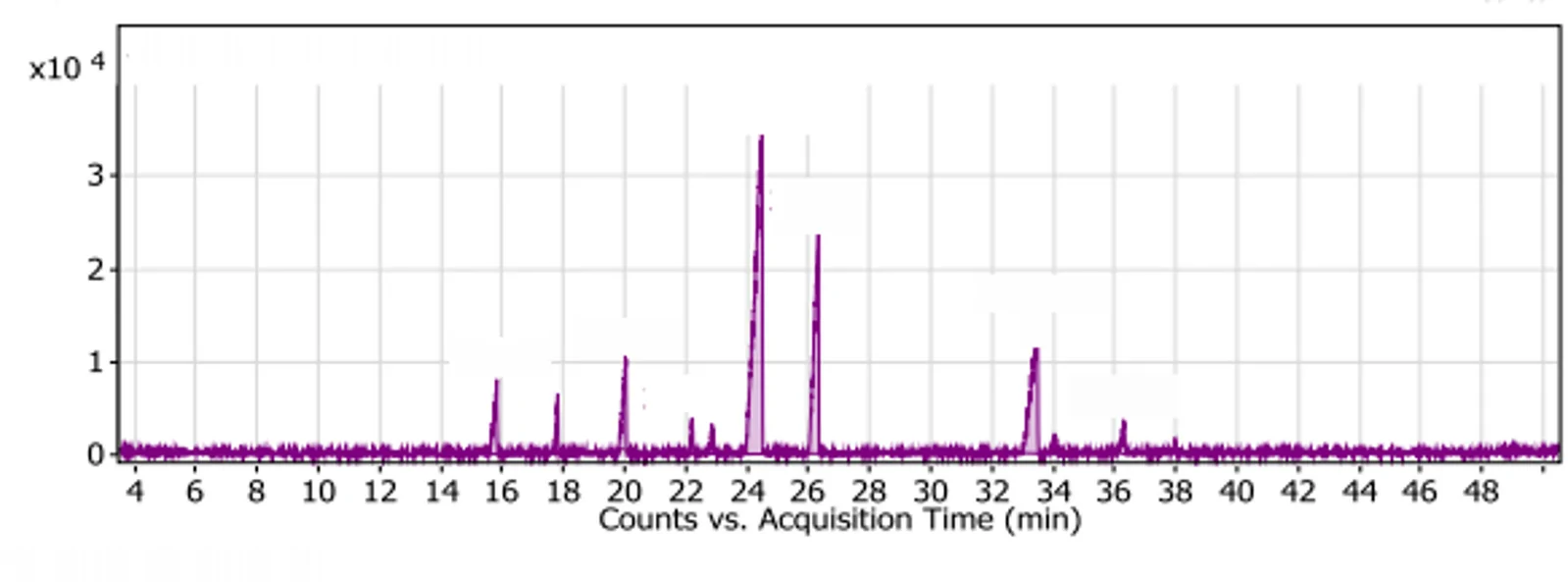
Essential oil constitutes from Azolla extract treatment (50%) at second harvest

**Fig. 8:**
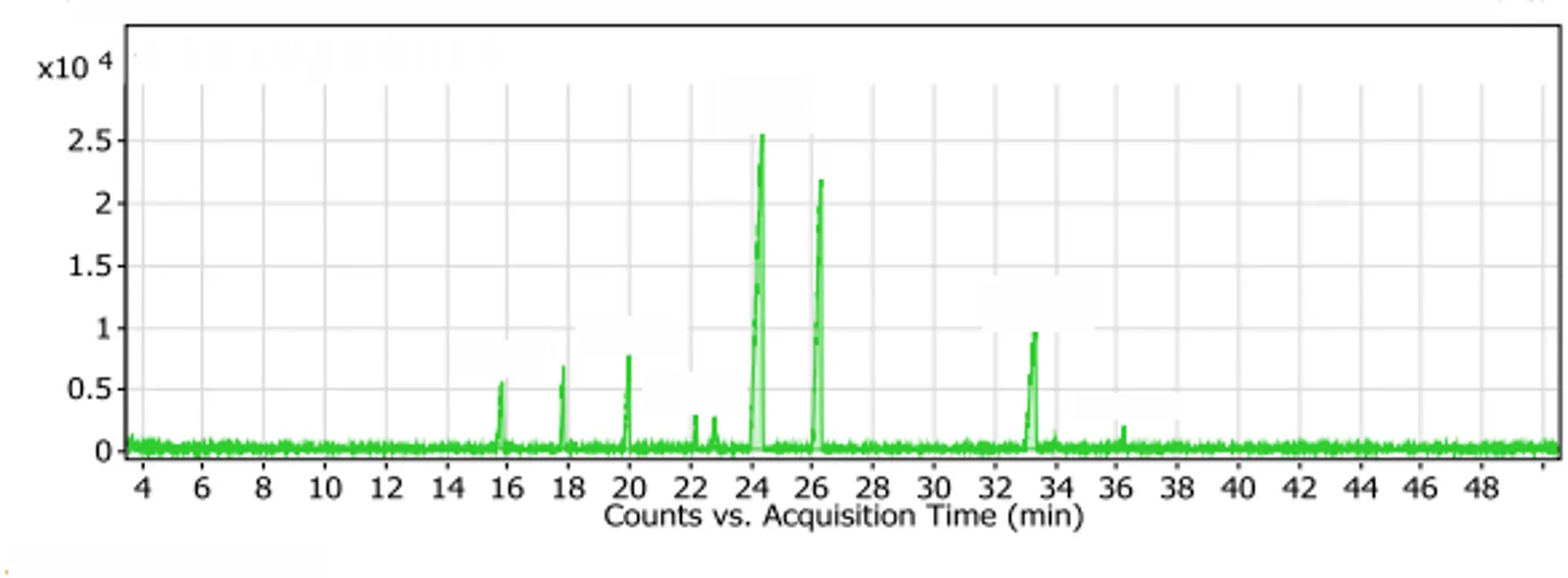
Essential oil constitutes from Azolla extract treatment (75%) at second harvest

**Fig. 9:**
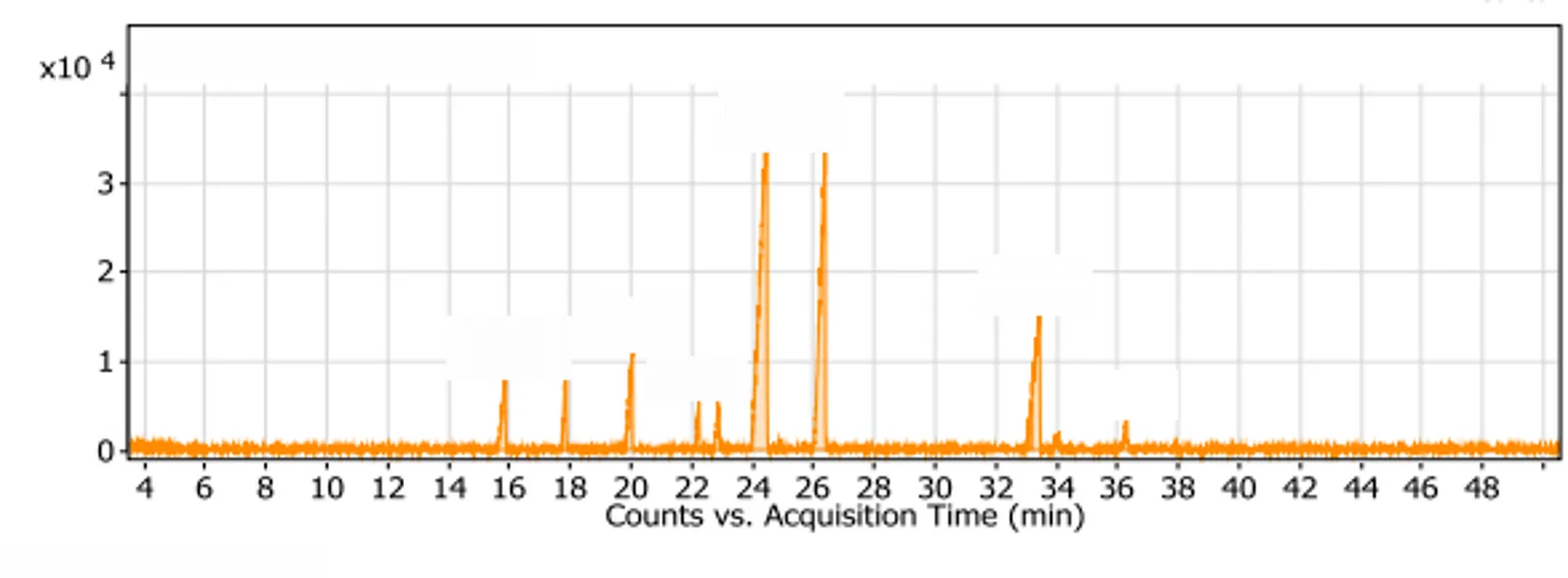
Essential oil constitutes from Moringa extract treatment (5%) at second harvest

**Fig. 10:**
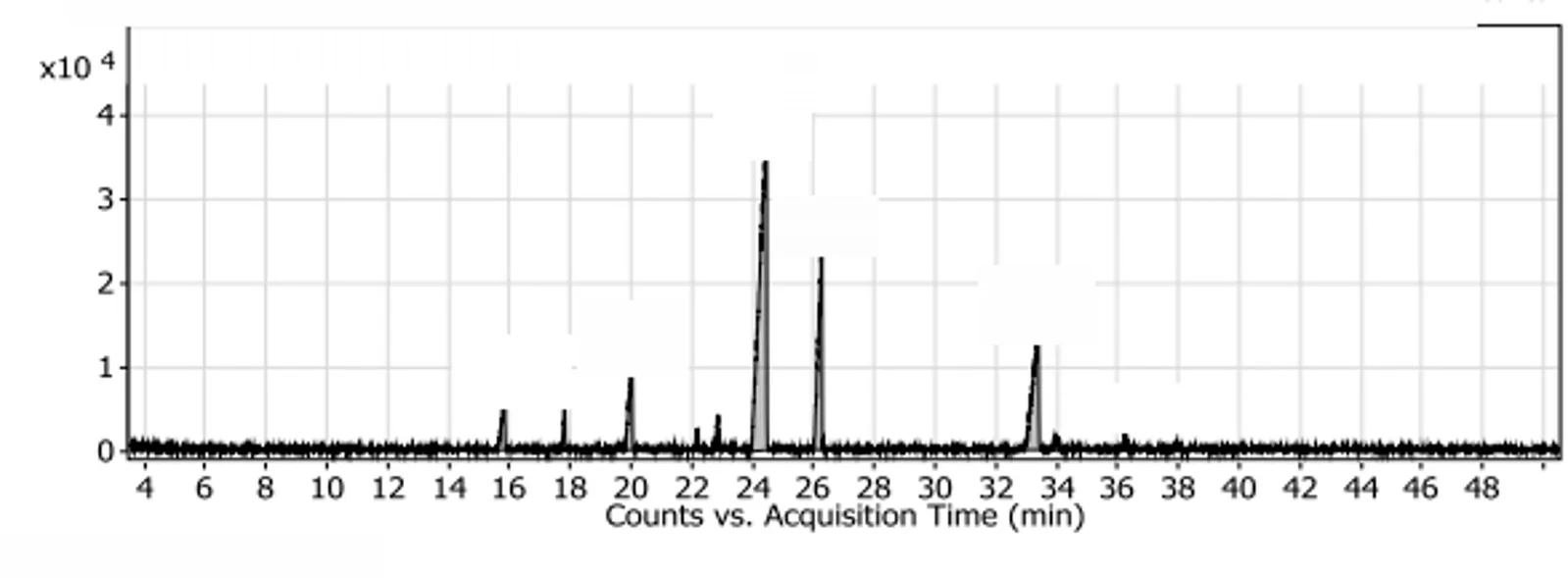
Essential oil constitutes from Moringa extract treatment (10%) at second harvest

Tables 7, 8 and 9 illustrate how applications and harvests influence the chemical composition of essential oils obtained from the aerial parts of geranium. Hydro-distilled essential oil components were quantitatively evaluated, and different variations in their chemical profiles were identified among the different applications and across the two harvesting (Figs. 1, 2, 3, 4, 5, 6, 7, 8, 9 and 10). Citronellol, geraniol and α-gurjunene were recognized as the predominant components. The minor constituents included α-pinene, myrcene, *cis*-β-ocimene, menthone, linalool acetate, germacrene D, phenethyl tiglate and α-humulene. All detected constituents were categorized into four classes: monoterpene hydrocarbons, oxygenated monoterpenes, sesquiterpene hydrocarbons, and oxygenated sesquiterpenes. Among these, oxygenated monoterpenes were the dominant group, while the other three classes occurred as minor fractions.

**Table 7:** Effect of the interaction treatments on essential oil constituents

No.	Constituents(mg plant^-1^)	RI	1^st^ harvest					2^nd^ harvest				
			Control	Azolla extract (%)		Moringa leaf extract (%)		Control	Azolla extract		Moringa leaf extract	
				50	75	5	10		50	75	5	10
1	α-Pinene	933	1.8 ± 0.3	0.8 ± 0.2	0.9 ± 0.1	1.1 ± 0.3	1.2 ± 0.3	2.4 ± 0.4	0.9 ± 0.5	1.1 ± 0.3	1.2 ± 0.3	1.4 ± 0.3
2	Myrcene	991	3.0 ± 0.5	10.4 ± 2.4	0.9±-/2	6.6 ± 1.1	10.8 ± 2.8	4.0 ± 0.8	11.7 ± 1.8	1.1 ± 0.2	7.2 ± 1.7	12.6 ± 1.7
3	cis-β-Ocimene	1040	22.8 ± 2.5	18.4 ± 2.9	29.7 ± 2.3	38.5 ± 3.6	24.0 ± 3.4	30.4 ± 1.7	20.7 ± 2.5	36.3 ± 2.6	42.0 ± 3.1	28.0 ± 1.4
4	Menthone	1154	27.6 ± 1.8	36.8 ± 3.5	38.7 ± 3.6	50.6 ± 3.9	51.6 ± 3.8	36.8 ± 1.3	41.4 ± 3.6	47.3 ± 3.2	55.2 ± 2.8	60.2 ± 2.6
5	Citronellol	1228	329.4 ± 5.7	413.6 ± 7.5	422.1 ± 6.8	497.2 ± 6.8	663.6 ± 7.3	439.2 ± 7.4	465.3 ± 8.4	515.9 ± 8.4	542.4 ± 5.7	774.2 ± 7.3
6	Linalool acetate	1257	7.2 ± 1.1	9.6 ± 1.7	11.7 ± 1.7	19.8 ± 2.3	13.2 ± 1.7	9.6 ± 1.1	10.8 ± 1.6	14.3 ± 1.6	21.6 ± 2.8	15.4 ± 1.6
7	Geraniol	1276	103.8 ± 2.6	152.8 ± 3.5	244.8 ± 4.7	287.1 ± 4.1	204.0 ± 3.7	138.4 ± 3.5	171.9 ± 3.7	299.2 ± 3.7	313.2 ± 4.9	238.0 ± 3.1
8	α-Gurjunene	1409	83.4 ± 2.1	129.6 ± 3.7	126.9 ± 3.7	159.5 ± 2.5	192.0 ± 2.6	111.2 ± 3.3	145.8 ± 3.7	155.1 ± 4.1	174.0 ± 3.3	224.0 ± 2.9
9	Germacrene D	1480	10.8 ± 1.2	11.2 ± 1.8	15.3 ± 1.4	24.2 ± 1.7	21.6 ± 1.4	14.4 ± 2.4	12.6 ± 2.1	18.7 ± 1.6	26.4 ± 1.6	25.2 ± 1.8
10	Phenethyl tiglate	1556	9.0 ± 1.5	13.6 ± 1.5	11.7 ± 2.8	15.4 ± 1.4	20.4 ± 1.5	12.0 ± 3.1	15.3 ± 0.9	14.3 ± 1.8	16.8 ± 2.1	23.8 ± 1.7
11	α-Humulene	1716	1.8 ± 0.7	4.0 ± 0.7	0.9 ± 0.3	1.1 ± 0.3	1.2 ± 0.3	2.4 ± 0.9	4.5 ± 0.7	1.1 ± 0.3	1.2 ± 0.4	1.4 ± 0.3
Chemical groups												
Monoterpene hydrocarbons (1–3)			27.6 ± 2.5	29.6 ± 2.5	31.5 ± 1.6	46.2 ± 2.8	36.0 ± 1.8	36.8 ± 1.6	33.3 ± 2.7	38.5 ± 2.5	50.4 ± 2.8	42.0 ± 2.6
Oxygenated monoterpene (4–7)			468.0 ± 6.2	612.8 ± 7/5	717.3 ± 8.3	854.7 ± 8.3	932.4 ± 9.3	624.0 ± 6.9	689.4 ± 7.4	876.7 ± 7.5	932.4 ± 9.4	1087.8 ± 10.5
Sesquiterpene hydrocarbons (8–9)			94.2 ± 1.8	140.8 ± 3.2	142.2 ± 4.2	183.7 ± 2.9	213.6 ± 2.8	125.6 ± 3.8	158.4 ± 2.6	173.8 ± 3.7	200.4 ± 3.5	249.2 ± 4.3
Oxygenated sesquiterpenes (10–11)			10.8 ± 1.1	17.6 ± 1.3	12.6 ± 1.8	16.5 ± 1.8	22.8 ± 1.5	14.4 ± 2.2	19.8 ± 2.4	15.4 ± 1.7	18.0 ± 2.7	26.6 ± 2,7

Major components (no 5, 7 and 8) ; minor components (no 1–4, 6 and 9–11) ; RI (retention index) Values are given as mean ± SD

**Table 8:** Effect of various applications or harvests on essential oil constituents

No.	Constituents(mg plant^-1^)	RI	Treatments					Harvests	
			Control	Azolla extract (%)		Moringa leaf extract (%)		1st	2^nd^
				50	75	5	10		
1	α-Pinene	933	2.1 ± 0.5	0.9 ± 0.3	1.0 ± 0.3	1.2 ± 0.2	1.3 ± 0.2	1.2 ± 0.3	1.4 ± 0.3
2	Myrcene	991	3.5 ± 0.9	11.1 ± 1.4	1.0 ± 0.2	6.9 ± 1.1	11.7 ± 1.4	6.3 ± 0.8	7.3 ± 0.9
3	*cis*-β-Ocimene	1040	26.6 ± 2.1	19.6 ± 2.3	33.0 ± 2.5	40.3 ± 2.4	26.0 ± 2.6	26.7 ± 0.8	31.5 ± 2.7
4	Menthone	1154	32.2 ± 1.9	39.1 ± 3.7	43.0 ± 3.2	52.9 ± 2.6	55.9 ± 5.6	41.1 ± 1.6	48.2 ± 2.8
5	Citronellol	1228	384.3 ± 4.8	439.5 ± 6.7	469.0 ± 4.7	519.8 ± 7.3	718.9 ± 7.4	465.2 ± 6.6	547.4 ± 5.7
6	Linalool acetate	1257	8.4 ± 1.1	10.2 ± 2.1	13.0 ± 2.1	20.7 ± 2.1	14.3 ± 2.4	12.3 ± 2.4	14.3 ± 2.5
7	Geraniol	1276	121.1 ± 3.5	162.4 ± 4.7	272.0 ± 3.9	300.2 ± 4.2	221.0 ± 4.1	198.5 ± 3.3	232.1 ± 4.8
8	α-Gurjunene	1409	97.3 ± 3.1	137.7 ± 3,5	141.0 ± 1.6	166.8 ± 2.7	208.0 ± 3.5	138.3 ± 3.7	162.0 ± 3.6
9	Germacrene D	1480	12.6 ± 1.7	11.9 ± 2.7	17.0 ± 1.2	25.3 ± 2.1	23.4 ± 1.7	16.6 ± 2.1	19.5 ± 2.7
10	Phenethyl tiglate	1556	10.5 ± 1.1	14.5 ± 2.8	13.0 ± 0.8	16.1 ± 2.1	22.1 ± 1.1	14.0 ± 1.8	16.4 ± 1.8
11	α-Humulene	1716	2.1 ± 0.9	4.3 ± 1.8	1.0 ± 0.2	1.2 ± 0.2	1.3 ± 0.3	1.8 ± 0.3	2.1 ± 0.5
Chemical groups									
Monoterpene hydrocarbons (1–3)			32.2 ± 2.7	31.5 ± 3.4	35.0 ± 2.4	48.3 ± 2.4	39.0 ± 2.4	34.2 ± 2.7	40.2 ± 2.4
Oxygenated monoterpene (4–7)			546.0 ± 6.5	651.1 ± 3.9	797.0 ± 6.9	893.6 ± 8.5	1010.1 ± 7.8	717.0 ± 7.9	842.1 ± 8.9
Sesquiterpene hydrocarbons (8–9)			109.9 ± 3.3	149.6 ± 4.3	158.0 ± 3.8	192.1 ± 3.1	231.4 ± 1.6	154.9 ± 3.5	181.5 ± 3.6
Oxygenated sesquiterpenes (10–11)			12.6 ± 2.4	18.7 ± 2.5	14.0 ± 1.6	17.3 ± 0.8	24.7 ± 1.4	16.1 ± 1.7	18.8 ± 2.5

Major components (no 5, 7 and 8) ; minor components (no 1–4, 6 and 9–11) ; RI (retention index) Values are given as mean ± SD

**Table 9:** Analysis of variance of essential oil constituents

No	Constituents	F values		
		Collections	Applications	Collections X Applications
1	.α-Pinene	8.3^**^	27.5^***^	1.2^ns^
2	Myrcene	27^***^	486.6^***^	2.2^ns^
3	.cis-β-Ocimene	251.2^***^	537.6^***^	10.7^***^
4	Menthone	4241.2^***^	6231.2^***^	92.3^***^
5	Citronellol	12891.6^***^	24828.6^***^	383.9^***^
6	Linalool acetate	318.5^***^	1369.4^***^	4.7^**^
7	Geraniol	4164.6^***^	16283.8^***^	128.9^***^
8	.α-Gurjunene	1148.3^***^	2719.6^***^	25.0^***^
9	Germacrene D	693.8^***^	2500.5^***^	14.8^***^
10	Phenethyl tiglate	155.2^***^	402.3^***^	3.8^*^
11	.α-Humulene	4.5^*^	63.5^***^	0.4^ns^
	Chemical groups			
Monoterpene hydrocarbons (1–3)		862.9^***^	901.7^***^	862.9^***^
Oxygenated monoterpene (4–7)		279656.6^***^	491721.9^***^	279656.6^***^
Sesquiterpene hydrocarbons (8–9)		9.0^**^	29.5^***^	9.0^**^
Oxygenated sesquiterpenes (10–11)		263.2^***^	601.2^***^	263.2^***^

*ns *non-significant

***, *P* ≤ 0.001; **, *P* ≤ 0.01; *, *P* ≤ 0.05

#### Effect of the interactions

Essential oil extracted from untreated plants at the second harvest exhibited the highest α-pinene concentration (2.4 mg plant^− 1^); while the maximum α-humulene content (4.5 mg plant^− 1^) was recorded in essential oil from plants treated with 50% Azolla extract during the second harvest. Essential oil obtained from plants treated with 5% Moringa extract during the second harvest exhibited the highest concentrations of *cis*-ß-ocimene (42 mg plant^− 1^), linalool acetate (21.6 mg plant^− 1^), geraniol (313.2 mg plant^− 1^), and total monoterpene hydrocarbons (50.4 mg plant^− 1^). Essential oil extracted from plants treated with 10% Moringa extract during the second harvest showed a marked increase in the accumulation of myrcene (12.6 mg plant^− 1^), menthone (60.2 mg plant^− 1^), citronellol (774.2 mg plant^− 1^), α-gurjunene (224 mg plant^− 1^), germacrene D (25.2 mg plant⁻¹), phenethyl tiglate (23.8 mg plant⁻¹), as well as total oxygenated monoterpenes (1087.8 mg plant^− 1^), sesquiterpene hydrocarbons (249.2 mg plant^− 1^), and oxygenated sesquiterpenes (26.6 mg plant^− 1^). It was noted that Compared with the control, treatment with 10% Moringa extract resulted in a pronounced enhancement of main components and major group. Citronellol, geraniol, α-gurjunene and oxygenated monoterpene group increased by 2.4, 3.3, 2.7 and 2.3 fold, respectively. The interaction effects between treatments and harvests (Table 7) induced highly significant variations (*P ≤ 0.001*) in most essential oil constituents and their chemical groups. In contrast, phenethyl tiglate showed a significant response at *P* ≤ 0.05, while α-pinene, myrcene, α-humulene, and sesquiterpene hydrocarbons were not significantly affected by the interaction.

#### Effect of the applications

The highest α-pinene concentration (2.1 mg plant^− 1^) was detected in the essential oil of untreated plants, whereas plants treated with 50% Azolla extract produced the maximum α-humulene content (4.3 mg plant^− 1^). Essential oil extracted from plants treated with 5% Moringa extract exhibited the greatest accumulation of *cis*-β-ocimene (40.3 mg plant^− 1^), linalool acetate (20.7 mg plant^− 1^), geraniol (300.2 mg plant^− 1^), germacrene D (25.3 mg plant^− 1^), and total monoterpene hydrocarbons (48.3 mg plant^− 1^). Plants treated with 10% Moringa extract produced essential oil rich in several volatile constituents, recording the highest concentrations of myrcene (11.7 mg plant^− 1^), menthone (55.9 mg plant^− 1^), citronellol (718.9 mg plant^− 1^), α-gurjunene (208 mg plant^− 1^), phenethyl tiglate (22.1 mg plant^− 1^), oxygenated monoterpenes (1010.1 mg plant^− 1^), sesquiterpene hydrocarbons (231.4 mg plant^− 1^), and oxygenated sesquiterpenes (24.7 mg plant^− 1^). Application treatments (Table 8) induced highly significant (*P ≤ 0.001*) variations in the composition and grouped fractions of the essential oil constituents.

#### Effect of the collections

Essential oil extracted from plants harvested during the second collection recorded the highest values for all identified constituents (Table 8), including α-pinene (1.4 mg plant^− 1^), myrcene (7.3 mg plant^− 1^), *cis*-ß-ocimene (31.5 mg plant^− 1^), menthone (48.2 mg plant^− 1^), citronellol (547.4 mg plant^− 1^), linalool acetate (14.3 mg plant^− 1^), geraniol (232.1 mg plant^− 1^), α-gurjunene (162 mg plant^− 1^), germacrene D (19.5 mg plant^− 1^), phenethyl tiglate (16.4 mg plant^− 1^), α-humulene (2.1 mg plant^− 1^), total monoterpene hydrocarbons (40.2 mg plant^− 1^), oxygenated monoterpenes (842.1 mg plant^− 1^), sesquiterpene hydrocarbons (181.5 mg plant^− 1^), and oxygenated sesquiterpenes (18.8 mg plant^− 1^). Harvests induced highly significant variations (*P ≤ 0.001*) in the different essential oil constituents and their grouped fractions. In contrast, α-pinene and sesquiterpene hydrocarbons showed moderately significant differences (*P ≤ 0.01*), whereas α-humulene responded significantly at *P* ≤ 0.05.

### Changes in total phenolic content in response to different applications across harvests

Total phenolic content was significantly influenced by the application of Azolla and Moringa extracts, harvest time, and their interaction (Table 10). Application of both extracts markedly increased total phenolic content compared with the untreated control plants. In general, phenolic accumulation was higher during the second harvest than the first harvest in both seasons. The maximum phenolic content values (16.6 and 19.3 mg g^− 1^) were obtained from plants treated with Moringa extract at 10% during the second harvest in the first and second seasons, respectively (increasing it by 1.5 and 1.6 fold during both seasons compared with control). Statistical analysis revealed that the variations in total phenolic content were highly significant (*P ≤ 0.001*) in response to various applications and harvests, whereas the interaction effect was significant (*P ≤ 0.05*) in the first season and non-significant in the second season.

**Table 10:** Effect of Azolla and Moringa extracts on the chemical components in both harvests

Collections	Applications(%)		Phenols(mg g^-1^)		Flavonoids (mg g^-1^)		IFRS(%)		Carbohydrates(%)		Protein (%)	
			Seasons									
			1^st^	2^nd^	1^st^	2^nd^	1^st^	2^nd^	1^st^	2^nd^	1^st^	2^nd^
First	Control		10.5 ± 2.1	13.0 ± 2.7	5.0 ± 0.9	6.8 ± 2.4	36.3 ± 3.7	34.1 ± 4.2	12.5 ± 3.5	14.0 ± 1.7	10.6 ± 1.1	11.9 ± 0.7
	Azolla	50	11.4 ± 2.3	13.3 ± 2.5	6.2 ± 1.1	7.5 ± 2.7	40.4 ± 2.7	43.2 ± 3.6	13.6 ± 2.6	14.8 ± 0.9	12.3 ± 1.8	13.8 ± 1.5
		75	11.9 ± 2.1	14.2 ± 1.7	7.1 ± 2.1	8.0 ± 2.7	43.8 ± 3.2	44.8 ± 3.8	14.9 ± 1.7	15.8 ± 4.3	15.0 ± 2.3	14.4 ± 0.8
	Moringa	5	12.2 ± 3.2	14.6 ± 3.7	7.9 ± 1.8	8.3 ± 0.8	44.9 ± 2.3	47.7 ± 2.5	15.2 ± 3.2	16.5 ± 1.7	16.3 ± 1.6	15.6 ± 1.7
		10	12.8 ± 2.8	15.4 ± 3.2	8.4 ± 2.3	8.9 ± 2.4	45.1 ± 3.6	55.7 ± 5,2	16.0 ± 0.8	17.0 ± 2.3	16.9 ± 3.1	18.1 ± 3.9
Overall first			11.8 ± 1.8	14.1 ± 2.8	6.9 ± 1.5	7.9 ± 3.1	42.1 ± 4.2	45.1 ± 3.5	14.4 ± 1.7	15.6 ± 3.6	14.2 ± 1.5	14.8 ± 2.2
Second	Control		12.3 ± 2.9	15.3 ± 1.7	4.7 ± 1.7	7.0 ± 1.1	41.0 ± 4.2	46.4 ± 2.7	14.7 ± 2.6	15.6 ± 2.8	12.5 ± 2.7	14.4 ± 0.6
	Azolla	50	12.8 ± 1.9	16.7 ± 2.7	6.8 ± 2.4	7.3 ± 1,6	41.6 ± 1.7	51.0 ± 6.3	15.7 ± 4.3	16.2 ± 1.4	15.0 ± 3.2	14.4 ± 1.5
		75	14.2 ± 4.4	17.5 ± 3.5	8.1 ± 1.5	8.8 ± 2.2	49.7 ± 4.6	53.4 ± 4.3	15.9 ± 2.2	17.0 ± 0.6	15.6 ± 2.7	15.6 ± 0.8
	Moringa	5	15.1 ± 3.8	18.6 ± 3.4	8.8 ± 1.7	9.4 ± 2.4	53.8 ± 5.2	56.8 ± 2.7	16.3 ± 3.7	18.6 ± 4.2	17.5 ± 2.4	16.3 ± 2.7
		10	16.6 ± 1.3	19.3 ± 4.2	9.4 ± 2.4	10.7 ± 3.1	62.4 ± 5.2	60.2 ± 4.3	17.2 ± 2.5	19.3 ± 4.1	18.8 ± 1.9	18.1 ± 0.5
Overall second			14.2 ± 3.3	17.5 ± 4.3	7.6 ± 1.7	8.6 ± 1.6	49.7 ± 2.6	53.6 ± 3.7	16.0 ± 2.1	17.3 ± 1.7	15.9 ± 2.6	15.8 ± 1.4
Overall Applications	Control		11.4 ± 2.5	14.2 ± 2.6	4.9 ± 1.6	6.9 ± 0.7	38.6 ± 3.7	40.2 ± 4.2	13.6 ± 4.3	14.8 ± 0.6	11.6 ± 3.2	13.2 ± 0.7
	Azolla	50	12.1 ± 3.5	15.0 ± 4.6	6.5 ± 0.4	7.4 ± 1.5	41.0 ± 2,1	47.1 ± 4.7	14.7 ± 2.8	15.5 ± 3.2	13.7 ± 2.6	14.1 ± 1.6
		75	13.1 ± 2.4	15.9 ± 2.1	7.6 ± 1.7	8.4 ± 2.4	46.8 ± 2.7	49.1 ± 3.2	15.4 ± 2.5	16.4 ± 2.9	15.3 ± 1.8	15.0 ± 0.7
	Moringa	5	13.7 ± 2.5	16.6 ± 3.5	8.4 ± 0.8	8.9 ± 0.7	49.3 ± 4.3	52.3 ± 4.9	15.8 ± 3.4	17.6 ± 1.4	16.9 ± 0.7	16.0 ± 0.4
		10	14.7 ± 1.8	17.4 ± 1.7	8.9 ± 1.6	9.8 ± 0.5	53.7 ± 2.7	57.9 ± 4.6	16.6 ± 2.8	18.2 ± 2.3	17.9 ± 1.1	18.1 ± 1.4
F values												
Collections			131.3^***^	171.0^***^	13.7^**^	10.8^**^	110.0^***^	264.0^***^	84.1^***^	13.8^***^	32.7^***^	19.2^***^
Applications			29.5^***^	19.2^***^	69.5^**^	21.1^**^	57.5^***^	125.6^***^	37.6^***^	7.2^***^	59.2^***^	56.6^***^
Collections x Applications			3.9^*^	1.4 ^ns^	2.0 ^ns^	2.4 ^ns^	14.2^***^	5.8^***^	2.5 ^ns^	0.2 ^ns^	4.4^*^	3.5^*^

*ns *non-significant

***, *P* ≤ 0.001; **, *P* ≤ 0.01; *, *P* ≤ 0.05; values are given as mean ± SD

### Effect of different applications on total flavonoid content across different harvests

Adding Azolla and Moringa extracts enhanced total flavonoid content compared with the control treatment, with greater accumulation recorded during the second harvest than the first (Table 10). The highest flavonoid contents (9.4 and 10.7 mg g⁻¹) were obtained from plants treated with Moringa extract at 10% during the second harvest in both seasons (this treatment enhanced total flavonoid content by 1.6- and 1.9-fold compared with the control during the first and second seasons, respectively). Total flavonoid content was significantly affected (*P ≤ 0.01*) by both application treatments and harvests, whereas the interaction between them was non-significant.

### Variations in free radical scavenging activity as affected by applications and harvests

Application of Azolla and Moringa extracts significantly enhanced free radical scavenging activity compared with the control treatment, with higher inhibition percentages recorded during the second harvest than the first (Table 10). The highest inhibition values (62.34 and 60.2%) were observed in plants treated with Moringa extract at 10% during the second harvest in both seasons **(**Free radical scavenging activity was 1.7- and 1.8-fold higher than the control in both seasons**).** Statistical analysis indicated that application treatments, harvest times, and their interaction had highly significant effects (*P ≤ 0.001*) on free radical scavenging activity.

### Effect of different applications and harvests on total carbohydrates

Application of Azolla and Moringa extracts markedly increased total carbohydrate accumulation in treated plants, with a clear superiority recorded at the second harvest compared to the first (Table 10). The highest total carbohydrate content (17.2% and 19.3% in the first and second seasons, respectively) was obtained from plants treated with Moringa extract at 10% during the second harvest. **(**Total carbohydrate content was 1.3-fold higher in plants treated with 10% moringa extract than in the control in both seasons**).** Statistical analysis showed highly significant effects of both treatment application and harvest stage (*P ≤ 0.001*), while the interaction effect between them was not statistically significant, indicating that each factor independently influenced carbohydrate accumulation.

### Effects of various applications and harvests time on crude protein

Application of Azolla and Moringa extracts significantly increased crude protein content compared with the control treatment, with consistently higher values recorded at the second harvest than at the first harvest (Table 10). The highest crude protein contents (18.8% and 18.1% in the first and second seasons, respectively) were obtained from plants treated with Moringa extract at 10% during the second harvest. (Crude protein content was 1.5 and 1.8 fold higher than the control in the first and second seasons**).** Statistical analysis revealed highly significant effects of both treatment application and harvest stage (*P ≤ 0.001*). In addition, the interaction between application treatments and harvest stage was significant (*P ≤ 0.05*), indicating that the response of crude protein accumulation varied depending on the combination of both factors.

### Changes in mineral content as affected by applications and harvests

The application of Azolla and Moringa extracts significantly enhanced the concentrations of N, P, and K in plant tissues compared with the control treatment. In general, higher N, P, and K contents were recorded at the second harvest than at the first harvest (Table 11). The highest nutrient contents were recorded in plants treated with 10% Moringa extract during the second harvest. N reached 3.1% and 2.9% in the first and second seasons, respectively, whereas P and K reached 0.5% and 2.5%, respectively, in both seasons. Relative to control, Moringa extract at 10% gave an increase in NPK contents. N increased by 1.5–1.8 fold in both seasons, whereas P and K contents increased by 2.5 fold and 1.5–1.7 fold, respectively. Statistical analysis showed highly significant effects of both application treatments and harvest stage (*P* ≤ 0.001). Moreover, the interaction between treatments and harvest stage was also significant (*P* ≤ 0.05).

**Table 11:** Effect of Azolla and Moringa extracts on the elemental contents in both harvests

Collections	Applications(%)		Elemental contents (%)					
			Nitrogen		Phosphorous		Potassium	
			Seasons					
			1^st^	2^nd^	1^st^	2^nd^	1^st^	2^nd^
First	Control		1.7 ± 0.3	1.9 ± 0.3	0.2 ± 0.1	0.2 ± 0.1	1.6 ± 0.2	1.7 ± 0.4
	Azolla	50	1.9 ± 0.4	2.2 ± 0.5	0.2 ± 0.1	0.3 ± 0.1	1.8 ± 0.4	1.8 ± 0.3
		75	2.4 ± 0.3	2.3 ± 0.3	0.3 ± 0.1	0.3 ± 0.1	1.8 ± 0.3	2.0 ± 0.5
	Moringa	5	2.6 ± 0.4	2.5 ± 0.2	0.4 ± 0.2	0.4 ± 0.2	2.0 ± 0.4	1.9 ± 0.4
		10	2.7 ± 0.3	2.8 ± 0.3	0.4 ± 0.2	0.4 ± 0.2	2.3 ± 0.3	2.1 ± 0.5
Overall first			2.3 ± 0.2	2.4 ± 0.3	0.3 ± 0.1	0.3 ± 0.1	1.9 ± 0.2	1.9 ± 0.3
Second	Control		2.0 ± 0.4	2.3 ± 0.3	0.3 ± 0.1	0.3 ± 0.1	1.9 ± 0.3	1.8 ± 0.4
	Azolla	50	2.4 ± 0.5	2.3 ± 0.3	0.3 ± 0.1	0.4 ± 0.1	2.1 ± 0.2	2.0 ± 0.3
		75	2.5 ± 0.3	2.5 ± 0.5	0.3 ± 0.1	0.4 ± 0.2	2.2 ± 0.4	2.3 ± 0.4
	Moringa	5	2.8 ± 0.3	2.6 ± 0.3	0.4 ± 0.1	0.4 ± 0.1	2.4 ± 0.3	2.4 ± 0.4
		10	3.1 ± 0.7	2.9 ± 0.3	0.5 ± 0.2	0.5 ± 0.2	2.5 ± 0.2	2.5 ± 0.4
Overall second			2.5 ± 0.5	2.5 ± 0.4	0.4 ± 0.2	0.4 ± 0.1	2.2 ± 0.4	2.2 ± 0.3
Overall Applications	Control		1.9 ± 0.2	2.1 ± 0.3	0.3 ± 0.1	0.3 ± 0.1	1.8 ± 0.3	1.8 ± 0.1
	Azolla	50	2.2 ± 0.4	2.3 ± 0.4	0.3 ± 0.1	0.4 ± 0.2	2.0 ± 0.5	1.9 ± 0.4
		75	2.5 ± 0.2	2.4 ± 0.5	0.3 ± 0.1	0.4 ± 0.1	2.0 ± 0.4	2.2 ± 0.3
	Moringa	5	2.7 ± 0.6	2.6 ± 0.4	0.4 ± 0.1	0.4 ± 0.1	2.2 ± 0.3	2.2 ± 0.4
		10	3.0 ± 0.5	2.9 ± 0.6	0.5 ± 0.2	0.5 ± 0.2	2.4 ± 0.4	2.3 ± 0.2
F values								
Collections			32.7^***^	19.2^***^	42.9^***^	98.0^***^	59.1^***^	59.1^***^
Applications			59.2^***^	56.6^***^	123.2^***^	67.3^***^	28.5^***^	23.9^***^
Collections x Applications			3.4^*^	3.5^*^	16.1^*^	6.1^*^	6.8^*^	4.3^*^

***, *P* ≤ 0.001; **, *P* ≤ 0.01; *,*P* ≤ 0.05; values are given as mean ± SD

### Modifications in mineral uptake driven by harvest and applications

Application of Azolla and Moringa extracts markedly enhanced N, P, and K uptake compared with the control treatment, with greater nutrient uptake recorded at the second harvest than at the first harvest (Table 12). The highest nutrient uptake was recorded in plants treated with 10% Moringa extract during the second harvest. N uptake reached 3.6 and 3.7 g plant⁻¹ in the first and second seasons, respectively. P uptake reached 0.5 and 2.5 g plant⁻¹ in both seasons, whereas K uptake reached 3.0 and 3.2 g plant⁻¹ in the first and second seasons, respectively. Relative to control, Moringa extract at 10% gave an increase in NPK uptakes. N increased by 3.1–3.6 fold in both seasons, whereas P and K uptakes increased by 5–25 fold and 2.9–3.3 fold, respectively. Statistical analysis revealed highly significant effects of both application treatments and harvest stage (*P* ≤ 0.001). Furthermore, the interaction between application treatments and harvest stage was significant (*P* ≤ 0.05).

**Table 12:** Effect of Azolla and Moringa extracts on the elemental uptakes in both harvests

Collections	Applications(%)		Elemental uptake (g plant^-1^)					
			Nitrogen		Phosphorous		Potassium	
			Seasons					
			1^st^	2^nd^	1^st^	2^nd^	1^st^	2^nd^
First	Control		1.0 ± 0.2	1.2 ± 0.1	0.1 ± 0.0	0.1 ± 0.0	0.9 ± 0.2	1.1 ± 0.3
	Azolla	50	1.3 ± 0.3	1.5 ± 0.3	0.1 ± 0.0	0.2 ± 0.1	1.2 ± 0.3	1.3 ± 0.2
		75	1.7 ± 0.3	1.8 ± 0.3	0.2 ± 0.1	0.2 ± 0.1	1.3 ± 0.3	1.6 ± 0.5
	Moringa	5	2.1 ± 0.2	2.2 ± 0.2	0.3 ± 0.1	0.4 ± 0.2	1.6 ± 0.4	1.7 ± 0.3
		10	2.5 ± 0.3	2.9 ± 0.5	0.4 ± 0.2	0.4 ± 0.1	2.1 ± 0.2	2.1 ± 0.3
Overall first			1.7 ± 0.3	1.9 ± 0.13	0.2 ± 0.1	0.3 ± 0.1	1.4 ± 0.2	1.6 ± 0.4
Second	Control		1.6 ± 0.4	2.0 ± 0.1	0.2 ± 0.1	1.0 ± 0.3	1.5 ± 0.3	1.6 ± 0.4
	Azolla	50	2.1 ± 0.2	2.2 ± 0.3	0.3 ± 0.1	1.3 ± 0.3	1.8 ± 0.4	1.9 ± 0.5
		75	2.4 ± 0.4	2.7 ± 0.4	0.3 ± 0.1	1.7 ± 0.2	2.1 ± 0.5	2.4 ± 0.4
	Moringa	5	2.9 ± 0.5	3.1 ± 0.5	0.4 ± 0.1	2.1 ± 0.3	2.5 ± 0.5	3.0 ± 0.6
		10	3.6 ± 0.4	3.7 ± 0.4	0.5 ± 0.2	2.5 ± 0.3	3.0 ± 0.6	3.2 ± 0.5
Overall second			2.5 ± 0.3	2.7 ± 0.4	0.3 ± 0.1	1.7 ± 0.4	2.2 ± 0.3	2.4 ± 0.3
Overall Applications	Control		1.3 ± 0.3	1.6 ± 0.1	0.2 ± 0.1	0.6 ± 0.2	1.2 ± 0.4	1.4 ± 0.3
	Azolla	50	1.7 ± 0.1	1.9 ± 0.2	0.2 ± 0.1	0.8 ± 0.2	1.5 ± 0.4	1.6 ± 0.2
		75	2.1 ± 0.6	2.3 ± 0.3	0.3 ± 0.1	1.0 ± 0.3	1.7 ± 0.1	2.0 ± 0.4
	Moringa	5	2.5 ± 0.4	2.7 ± 0.5	0.4 ± 0.2	1.3 ± 0.4	2.1 ± 0.4	2.4 ± 0.3
		10	3.1 ± 0.5	3.3 ± 0.5	0.5 ± 0.2	1.5 ± 0.5	2.6 ± 0.5	2.7 ± 0.5
F values								
Collections			331.8 ^***^	249.5^***^	16.5^***^	33.9^***^	319.2^***^	301.1^***^
Applications			209.9 ^***^	153.2^***^	16.5^***^	12.0^***^	123.0^***^	96.3^***^
Collections x Applications			3.8 ^*^	0.6^*^	0.5^*^	0.7^*^	2.4^*^	7.3^*^

***, *P* ≤ 0.001; *, *P* ≤ 0.05; values are given as mean ± SD

## Discussion

Azolla and Moringa extracts significantly improved the growth and chemical composition of geranium plants grown in sandy soil.

The positive response of geranium plants to Azolla application may be attributed to its multifunctional role as a natural biostimulant. Through its symbiotic association with *Anabaena azollae*, Azolla enhances N availability, while its decomposition enriches the soil with organic matter and essential macro- and micronutrients, thereby improving soil fertility and nutrient uptake, particularly in sandy soils characterized by low fertility and poor water-holding capacity [46]. In addition, Azolla contains natural phytohormones, including auxins, gibberellins, and cytokinins, which stimulate cell division, growth, and metabolic activity. It also enhances rhizosphere microbial activity and antioxidant defense, contributing to improved physiological performance and greater tolerance to environmental stress [47]. Collectively, these effects promote vegetative growth and productivity, consistent with previous studies reporting improved soil properties, nutrient uptake, and biomass production following Azolla application [48]. Similar responses have been observed in cardoon, where Azolla significantly enhanced plant growth and biomass accumulation [49], and increased chlorophyll content and dry matter production, leading to improved overall productivity [50].

The increase in photosynthetic pigments observed after Azolla application is consistent with the enhanced nutritional status and physiological performance described above. Higher chlorophyll and carotenoid contents indicate improved photosynthetic capacity, which agrees with previous studies on jojoba and cardoon, where Azolla extract significantly increased chlorophyll *a* and chlorophyll *b* contents, particularly following foliar application, resulting in enhanced photosynthetic efficiency and plant performance [47–49, 51].

The enhancement of essential oil yield and terpene accumulation may be attributed to the improved physiological status and photosynthetic performance induced by Azolla. Enhanced carbon assimilation and metabolic activity likely promoted greater carbon allocation toward terpene biosynthesis, resulting in improved essential oil yield and composition. Similar findings were reported in basil, where Azolla treatments increased essential oil yield and the relative abundance of major monoterpenes and sesquiterpenes [49, 52].

The higher phenolic and flavonoid contents observed in the present study indicate stimulation of secondary metabolism, particularly the phenylpropanoid pathway, leading to enhanced antioxidant capacity. Hassan [53] reported that nutrient starvation stimulated the accumulation of phenolic compounds, flavonoids, and anthocyanins in *Azolla*, accompanied by increased PAL activity and antioxidant potential. Likewise, Cannavò [54] demonstrated that environmental stress enhanced phenolic metabolism in *Azolla*, whereas Al-Huqail [55] and Zeid [56] showed that Azolla-based biostimulants strengthened antioxidant defense systems and improved plant tolerance to salinity and heavy-metal stress.

The increased carbohydrate and protein contents observed in the present study are likely associated with the improved photosynthetic efficiency and N metabolism promoted by Azolla application. Through its symbiotic association with *Anabaena azollae*, Azolla enhances nitrogen availability, thereby supporting amino acid and protein synthesis, while its supply of essential nutrients and natural cytokinins promotes carbon assimilation and overall metabolic activity [48]. Similar improvements in carbon assimilation, nitrogen metabolism, and osmotic adjustment have been reported in wheat treated with *Azolla* extract under salinity stress, resulting in enhanced growth and stress tolerance [55]. These findings further support the role of Azolla-based biostimulants in improving primary metabolism under adverse environmental conditions.

The higher uptake of N, P, and K observed in Azolla-treated plants reflects the improved soil fertility and nutrient acquisition associated with Azolla application, together with the greater biomass production achieved in treated plants. Comparable improvements in nutrient uptake and utilization have also been reported in wheat and maize grown under salinity and heavy-metal stress following Azolla application [48, 55, 56].

Moringa extract enhanced plant growth through its rich composition of natural biostimulants, nutrients, and bioactive compounds [19, 57]. It contains high concentrations of cytokinins, particularly zeatin, which stimulates cell division, shoot proliferation, and delay leaf senescence, while its macro- and micronutrients, amino acids, vitamins, and antioxidants improve photosynthesis, protein synthesis, and overall metabolic activity. Collectively, these effects promote root and shoot development and improve plant productivity [58, 59]. Accordingly, the greater biomass production observed in geranium is likely associated with enhanced physiological performance and photosynthetic activity. The enhanced biomass production observed in geranium is therefore associated with improved physiological performance and photosynthetic activity. Similar responses have been reported in sacred basil [60], tulsi [61], and coriander [62].

The increase in photosynthetic pigments following Moringa application is consistent with the improved physiological status described above. Higher chlorophyll and carotenoid contents indicate enhanced photosynthetic capacity, which may result from the combined effects of zeatin, mineral nutrients, and antioxidant compounds that maintain chloroplast integrity and delay chlorophyll degradation [63–69]. Similar improvements have been reported by Rady [57], Aslam [65] and Ashraf [70].

Moringa extract also enhanced essential oil yield and composition, probably through its stimulatory effects on photosynthesis and secondary metabolism. Improved carbon assimilation and metabolic activity likely increased the availability of precursors required for terpene biosynthesis, resulting in greater essential oil accumulation and improved composition [63, 71]. Similar responses have been reported in geranium [72], *Ocimum gratissimum* [73], *Artemisia annua* [74], and palmarosa [75].

The higher phenolic and flavonoid contents, together with the enhanced antioxidant activity observed in the present study, indicate stimulation of secondary metabolism and antioxidant defense. These responses may be associated with the bioactive constituents of Moringa leaves, including phenolics, flavonoids, vitamins, minerals, and phytohormones, which promote the biosynthesis and accumulation of antioxidant metabolites [63–65]. Similar increases in phenolic compounds, flavonoids, and antioxidant capacity have been reported [76] in coriander [77], radish [70], spinach [65], and other crops treated with Moringa extract.

The increased carbohydrate and protein contents reflect the improved photosynthetic efficiency and nitrogen metabolism promoted by Moringa extract. Comparable increases in carbohydrate accumulation and protein synthesis have been reported following Moringa application in several crops [35, 63, 78, 79].

The higher concentrations and uptake of N, P, and K observed in geranium plants treated with Moringa extract are consistent with its positive effects on nutrient availability, root growth, and nutrient assimilation. Improved biomass production further contributed to greater nutrient accumulation in plant tissues. Similar improvements in mineral nutrient uptake have been reported in rocket, pear, mandarin, and other crops following Moringa extract application [57, 63, 76, 80, 81].

The present findings are consistent with recent studies demonstrating that plant extract-based biostimulants enhance plant growth, biomass accumulation, photosynthetic pigments, nutrient uptake, antioxidant capacity, and secondary metabolite production through multiple physiological and biochemical mechanisms [82–85]. These biostimulants also improve water status, nutrient use efficiency, soil health, crop quality, and tolerance to abiotic stresses while reducing oxidative damage and reliance on synthetic agricultural inputs, thereby promoting sustainable crop production [86, 87].

Successive harvesting acts as a repeated defoliation stress that triggers physiological and metabolic reprogramming in aromatic plants. Cutting stimulates the activation of dormant buds and enhances regrowth through increased cell division and expansion, supported by improved mobilization of stored carbohydrates and nutrients. This regrowth phase is associated with upregulation of photosynthetic activity, leading to higher chlorophyll content and increased carbon assimilation. Enhanced assimilate production promotes nutrient uptake (N, P, and K) and protein synthesis, supporting rapid biomass recovery. At the same time, repeated harvesting induces mild stress signaling, which activates secondary metabolism pathways, resulting in greater accumulation of phenolic compounds, flavonoids, and essential oil constituents. Overall, these physiological adjustments explain the observed changes in biomass, essential oil yield, and phytochemical composition under multiple harvest systems [27, 28].

The findings of the present study suggest that application of Azolla and Moringa extracts offers a promising and sustainable approach to improving geranium growth, productivity and essential oil yield under sandy soil conditions. These natural biostimulants may support the cultivation of geranium in newly reclaimed and desert lands in Egypt and contribute to more sustainable production systems. The observed improvements in plant growth and essential oil productivity may also provide potential economic benefits by reducing reliance on synthetic agrochemicals, although further large-scale field and economic studies are needed to confirm their commercial feasibility. In addition, the enhanced essential oil yield and composition observed in this study may improve the quality and potential applications of geranium essential oil, while further research is required to verify its industrial applicability.

## Conclusion

Azolla and Moringa extracts are effective natural biostimulants for improving the growth, physiological performance, nutrient uptake, and essential oil production of geranium grown in sandy soil. Their application enhanced vegetative growth, biomass accumulation, photosynthetic pigments, phenolic and flavonoid contents, antioxidant activity, and N, P, and K uptake, resulting in higher essential oil yield and quality across successive harvests. Among the tested treatments, the higher concentration of Moringa extract produced the greatest improvements. These findings highlight the potential of Azolla and Moringa extracts as sustainable alternatives to synthetic inputs for enhancing geranium production and essential oil quality in newly reclaimed and desert lands.

## Data Availability

The datasets used and/or analyzed during the current study are available from the corresponding author on responsible request.
